# Local economies and household spacing in early chiefdom communities

**DOI:** 10.1371/journal.pone.0252532

**Published:** 2021-05-27

**Authors:** C. Adam Berrey, Robert D. Drennan, Christian E. Peterson

**Affiliations:** 1 Department of Anthropology, Universidad de los Andes, Bogotá, Colombia; 2 Center for Comparative Archaeology, Department of Anthropology, University of Pittsburgh, Pittsburgh, Pennsylvania, United States of America; 3 Department of Anthropology, University of Hawai’i at Mānoa, Honolulu, Hawai’i, United States of America; Utah State University, UNITED STATES

## Abstract

Archaeological research has by now revealed a great deal of variation in the way early complex societies, or chiefdoms, developed. This variation is widely recognized, but our understanding of the forces that produced it remains relatively undeveloped. This paper takes aim at such understanding by exploring variation in the local economies of six early chiefdoms; it considers what implications this variation had for trajectories of chiefdom development, as well as the source of that variation. Economic exchange is a primary form of local interaction in all societies. Because of distance-interaction principles, closer household spacing within local communities facilitated more frequent interaction and thus encouraged productive differentiation, economic interdependence, and the development of well-integrated local economies. Well-integrated local economies, in turn, provided ready opportunities for aspiring leaders to accumulate wealth and fund political economies, and pursuit of these opportunities led to societies with leaders whose power had a direct economic base. Wider household spacing, on the other hand, impeded interaction and the development of well-integrated local economies. In such contexts, aspiring leaders were able to turn to ritual and religion as a base of social power. Even when well-integrated local economies offered opportunities for wealth accumulation and a ready source of funding for political economies, these opportunities were not always taken advantage of. That variation in the shapes of early chiefdoms can be traced back to patterns of household spacing highlights the importance of settlement and interaction in explaining not just chiefdom development, but societal change more generally.

## Introduction

Between 7000 and 1000 years ago, regional scale social formations integrating thousands of people emerged repeatedly and independently in numerous parts of the world. These early complex societies, loosely referred to as chiefdoms, were often (but not always) organized around political, productive, prestige, wealth, and/or ritual differentiation. Some of these forms of differentiation developed more strongly in some chiefdoms than in others. They combined in different ways to produce varied systems of chiefdom organization. The comparative exploration of the factors that shaped this variability is a powerful empirical approach to understanding not just chiefdom dynamics, but the dynamics of societal change more generally [1–3].

This paper focuses on variation in the local economy, by which we mean the production and distribution of goods used in daily life by the bulk of any population. These goods would comprise primarily subsistence and non-subsistence utilitarian items, such as clothing, containers, furnishings, and tools used for cutting, sewing, cultivating, and many other domestic activities. The production and distribution of highly elaborate ritual, sumptuary, and luxury goods have often been the focus of archaeological research, but such goods are not typically part of the local economy, as they are very often made of exotic materials and/or for export to other regions. These goods are by no means unimportant, but they circulate in relatively small quantities and are used by a very small proportion of any population. In the modern world, for example, luxury goods account for only a fraction of one percent of total production [4,5]. In the Neolithic, they were usually important for their symbolic and prestige value rather than for their strictly economic value. Goods used in substantial quantities by the majority of prehistoric populations were of necessity produced locally, given the limitations of prehistoric transport technology [6,7].

Because the local economy consisted primarily of utilitarian goods, the notion is not unrelated to the distinction made by Hayden [8] between practical and prestige technologies. Hayden’s practical technologies are what is primarily produced and circulated in what we call the local economy, although the local economy can also include some items that are not practical in the strictest sense. The local economy overlaps substantially with what Hirth [9,10] has labeled the domestic economy and, like it, is based on household production; here, though, we have tried to exclude the production of luxury goods for long-distance exchange. D’Altroy and Earle’s [11] distinction between staple and wealth finance also separates luxury goods from goods of practical use, but D’Altroy and Earle focus on elite finance, or funding a political economy. The local economy can have implications for funding political economies (as we discuss further below), but its fundamental role is provisioning a population with the basic necessities of daily life. The local economy thus provides the economic foundation for all aspects of society, with implications for general well-being, inequality, political organization, and more.

In order for any human society to survive, its local economy must be organized so as to function successfully, but this can be accomplished in a variety of ways. One key way in which local economies can vary is in how much interaction and interdependence they entail. The earliest form of economic interdependence was surely the kind of food sharing between families that is well documented for foraging societies [12,13]. With the Neolithic came substantial, though variable, increases in interdependence, and in the differentiation of household productive activities upon which such interdependence was based. This reorganization of productive activities across households is often discussed under the heading of *craft specialization*, but we will refer to it as *productive differentiation* [2]. We do this so as to include both subsistence and craft production, and to include levels of inter-household differences in productive activities that might be too low to satisfy the criteria of some definitions of *specialization* [14].

A web of local interdependence born from the frequent exchange of commonly used goods (i.e. the local economy) laced Neolithic households together in especially important ways, and this early interdependence was the root from which the economic interconnectedness of urban life eventually grew [15,16]. Agriculture, of course, entailed considerable changes in the scheduling of subsistence activities, and productive differentiation layered on more complexity in how activities were organized. As the exchange of goods inherent in economic interdependence increased, existing social relationships often were modified and new ones created. These changes in economic activity patterns would frequently have rippled out into ritual and political arenas. Variation in the degree of integration in local economies created or impeded different opportunities for the mobilization of resources for public purposes in the political economies that grew with the emergence of chiefdoms. Exploring this variation is thus vital to the critical effort of understanding broader patterns of variation in chiefdom trajectories.

This paper explores variation in the local economies of six early chiefdom communities, specifically as it relates to the degree of productive differentiation, economic interdependence, and household spacing within local communities. In the course of this exploration we develop a technique for measuring the degree of productive differentiation, discuss how those economies connected (or did not connect) to the political economies of early chiefdoms, examine how distance-interaction principles and community layout impacted economic interdependence, and consider how community layout came to vary in the first place. We adopt a perspective that is global in scope, drawing on case studies from the Alto Magdalena (Colombia), the Barinas region (Venezuela), the Valley of Oaxaca (Mexico), the Western Liao Valley (China), the Anatolia Plain (Turkey), and the Middle Niger Delta (Mali). We begin by using archaeologically recovered household artifact assemblages to assess productive differentiation in each of these six cases, for which household spacing can be approximated from archaeologically established residential densities. We then explore the implications of household spacing for interaction using abstract examples based on contemporary villages. Finally, we relate the variation in productive differentiation observed in the archaeological cases to the distance-interaction principles revealed in the abstract examples.

## Exploring variation in the local economies of early chiefdoms

Economic interdependence is not directly observable in the archaeological record, so analytical approaches must be explicitly developed to identify it. The approach of most relevance here focuses on differences in the productive activities of individual households, as these are the differences that lead to the frequent exchange of basic goods and thus economic interdependence between households. The productive activities of individual households are most strongly reflected archaeologically in household artifact assemblages, especially in the tools used for different activities and the resulting production debris. Detecting such differences requires detailed data on the artifact assemblages of distinct households in a settlement [17], not just overall characterizations of the artifacts at a site (see *Methodological Implications* below).

We examine the local economies of six chiefdom communities by delineating patterns of variation in the artifact assemblages of different households using nonmetric multidimensional scaling analysis. The essential result of multidimensional scaling is a configuration of points, each one of which (in this analysis) represents a household assemblage. Points that lie close to each other in the configuration represent households whose artifact assemblages are similar, while household assemblages that are very different from each other are represented by points that lie far apart in the scaling configuration. A scaling configuration thus comprises a graphical presentation of the patterns of similarities between household artifact assemblages. Similarities between the assemblages were measured with Euclidean distance calculated on standardized variables. So as to focus attention as clearly as possible on productive differentiation (in contrast to other forms of inter-household differentiation), the variables used were proportions and ratios of artifacts related to productive activities; artifacts commonly thought to relate to prestige or wealth, such as luxury or ornamental items, were omitted from the analysis. For this reason, the scaling configurations presented here differ from those published previously for some of these same cases [1,18]. Below we present summary interpretations of each configuration and comparisons between them; lists of variables and complete results are available in the supporting information (S1 Text, S1 and S2 Tables).

## Productive differentiation in four regions

The Valley of Oaxaca (Mexico) provides an especially well-documented context for exploring productive differentiation in early farming villages. San José Mogote, the well-known regional center during Early and Middle Formative times (1500–500 BCE), with a complex of elaborate ceremonial and elite residential architecture [19,20], would be a logical place to examine productive differentiation and local economic interaction in this region. The sample of household artifact assemblages available for analysis, however, is complicated by the presence of households focused on the production of luxury goods for long-distance exchange, which interferes with an examination of the local economy.

**Fig 1:**
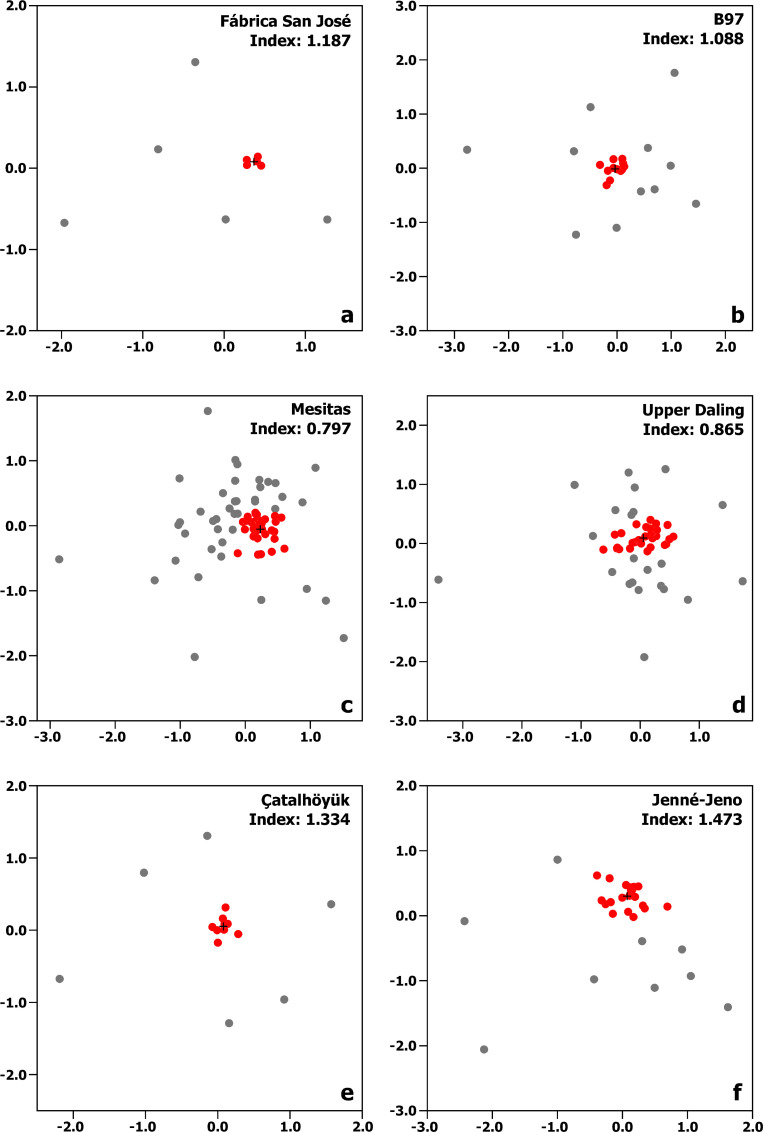
Multidimensional scalings of household artifact assemblages. a) Fábrica San José (Oaxaca), b) B97 (Barinas), c) Mesitas (Alto Magdalena), d) Upper Daling (Western Liao), e) Çatalhöyük (Anatolia), and f) Jenné-Jeno (Middle Niger). Red points correspond to the central cluster of ordinary households in each configuration; black cross corresponds to the centroid of each central cluster. Index value corresponds to the measure of productive differentiation discussed in the text.

The contemporaneous Middle Formative satellite village of Fábrica San José, about 5 km to the northeast of San José Mogote, provides a sample of 10 households that, while small, includes most of the households in the community; it thus provides a comprehensive view of productive differentiation within a Oaxaca village [21]. A multidimensional scaling of the artifact assemblages from these households shows a tight cluster of five ordinary households whose assemblages indicate a set of basic domestic daily activities and little else (the red points in Fig 1A). The close spacing of the points representing these households reflects the very strong homogeneity of their assemblages. Five other households have assemblages that are quite different, setting them apart from this tight cluster in the multidimensional space. These households are distinguished by high proportions of flaked stone, obsidian, shell, mica, polishing pebbles, ochre, sherd disks, bone needles, bone awls, bone gouges, and/or sherds from vessels used to collect salt. These artifacts relate to assorted productive activities in which these households engaged more intensively than those in the central cluster.

The five households set off from the central cluster are also distant from each other, a product of the fact that each has its own unique combination of artifacts. In some instances, specific productive activities are suggested (salt-making, sewing, leather-working, pottery making), while in others an abundance of particular artifacts shows a difference in productive activities, even though specific activities cannot be identified. Household artifact assemblages at this small village clearly indicate some degree of productive differentiation—enough to involve a certain degree of economic interdependence between households—although it does not seem likely that any household had completely abandoned food production or put its livelihood entirely in the hands of other households.

The characteristic of spatial patterning in the Fábrica San José scaling that is most relevant for present purposes is the degree to which outliers stand apart from the central cluster of more ordinary household assemblages. In a community with little or no productive differentiation, household artifact assemblages would resemble each other strongly but they would not be identical; they would differ from each other in a myriad of weak and trivial ways. In a multidimensional scaling configuration, these small differences would loom large, causing households to spread apart from each other across much of the scaling space. If a few households began to focus more of their energy on certain productive activities, their artifact assemblages would begin to differ more strongly from those of ordinary households, and they would increasingly take on the character of outliers in the scaling configuration. As their activities came to differentiate them more strongly, the outlying points that represent them in the scaling would move farther from ordinary households. For this to happen within the confines of scaling space, the points representing ordinary households will draw closer together into a tighter cluster. The stronger differentiation of a few households creates the tight clustering of ordinary households that more clearly reflects the genuine triviality of the differences between their assemblages. We would expect strongly differentiated households to be few in number in a population largely dedicated to subsistence production. (This goes not just for the Neolithic: as recently as 1600 CE some 50–70% of the European labor force were farmers [22].) In scalings of household assemblages, the early development of productive differentiation is thus most clearly seen in increasing distances between more specialized households and ordinary farming households.

The Barinas region in the western llanos of Venezuela is another context where available data permit an exploration of productive differentiation in early farming villages [23,24]. The regional center of El Gaván was by far the largest of these villages during Late Gaván times (550–1000 CE), but the smaller satellite community of B97, less than 0.5 km to the southwest of El Gaván, provides a better opportunity for assessing the full range of productive differentiation in the local economy, uncomplicated by the defensive and long-distance exchange activities that took place at the regional center. Excavation units scattered across the site provide 23 domestic artifact assemblages from different locations [25]. As at Fábrica San José, a multidimensional scaling reveals a dense core of highly similar assemblages that reflect ordinary domestic activities (Fig 1B). Other assemblages are spread at increasing distances from the core by high proportions of lithic artifacts, utilized flakes, reused flakes, grinding stones, axes, kiln wasters, polishing pebbles, sherd disks, and reworked, grooved, and notched sherds.

For Fábrica San José, the distinction between the central cluster and distant outliers is unmistakable; no analysis is needed to distinguish between these two groups. For B97, however, the boundary of the central cluster is less clear. Distinguishing the members of the dense core from the sparser distribution of points around it thus calls for a more systematic approach to spatial clustering that resolves the quandary of purely subjective definitions of cluster boundaries, several of which may be equally plausible. Spatial clustering by *k*-means provides just such an approach [26], one that does not remove subjective judgment entirely (since the number of clusters to be found must be specified by the analyst) but systematizes the subjectivity involved. A six-cluster *k*-means solution defines the central cluster of B97 well—better than solutions with any other number of clusters. The red points in Fig 1B are the members of the cluster in this six-cluster solution. In just the same way, the red points in Fig 1A are the members of the principal cluster in a six-cluster *k*-means analysis of the Fábrica San José configuration. (The *k*-means analysis simply replicated subjective judgment in defining that central cluster, but was carried out for the sake of systematic comparison.)

We have previously used the mean Euclidean distance between assemblages divided by the number of variables as a measure of the strength of household differentiation [18,27], but here we adopt a more robust approach based on the centroid of the principal cluster as identified by *k*-means analysis (a point in the configuration space whose X and Y coordinates are the mean X and Y coordinates of all the points within that cluster). These principal cluster centroids are shown in Fig 1A and 1B and form the basis for quantifying the degree to which households that are not part of the principal cluster are separated from it. The median distance from the principal cluster centroid to the points outside the cluster (in the arbitrary units of the configuration coordinate system) is 1.187 for Fábrica San José and 1.088 for B97. In the Fábrica San José configuration, the points outside the central cluster are strong outliers—they are, in other words, located far from the cluster centroid—and this produces the high median distance observed. For B97, some points lie far outside the principal cluster but others are close to it, producing a lower median distance that reflects a slightly weaker separation between cluster points and outliers. Because distances between points in these scalings reflect differences in assemblages of artifacts used in productive activities, the lower median for B97 reveals somewhat weaker productive differentiation than at Fábrica San José. Productive differentiation, then, is slightly less developed in the local economy of B97 than at Fábrica San José. Compared with the other regions we will discuss below, however, both Oaxaca and Barinas have relatively high degrees of economic interdependence in their local economies.

In the steep mountains of the Alto Magdalena (Colombia), a somewhat different pattern of productive differentiation appears in a chiefdom context. During the Regional Classic period (1–900 CE), numerous elaborate tombs accompanied by monumental sculpture appeared at a number of locations, heralding the emergence of hierarchical social organization. The most impressive and well-known of these occurred at Mesitas, near the modern town of San Agustín [1,28,29]. Between and around these tombs were 75 households for which artifact assemblage data are available [30].

A multidimensional scaling of the 75 household artifact assemblages at Mesitas reveals a pattern that is both similar to and different from that seen for Fábrica San José (Fig 1C). As in the Fábrica San José scaling, some of the Mesitas households cluster very tightly together in one region of the configuration space. Unlike the Fábrica San José scaling, however, this cluster, while unmistakable, is very difficult to delineate with precision. The density of points is very high at the core of the cluster, and gradually declines as distance from that core increases; only a few points stand off a bit more clearly from the others at the edges of the configuration. At the extreme left of the configuration is an artifact assemblage that contains higher proportions of flaked stone tools (especially of basalt), utilized flakes, scrapers, polishing stones, and sherd disks. Toward the upper and lower extremes of Fig 1C are households with higher proportions of flaked stone tools (especially of chert), scrapers, debitage, grinding stones, adzes, axes, chisels, kiln wasters, and/or cylindrical hammers. No household has everything on this list, and these items do occur in assemblages of the core cluster as well. Moreover, none of the households near the edges of the configuration contain these artifacts in proportions that differ as strongly from ordinary households as is the case in the Fábrica San José scaling.

The gradually declining density of points away from the core of the Mesitas scaling means the separation between a central cluster and outliers is even less distinct than at B97. The red points in Fig 1C are the members of the principal cluster as defined by a seven-cluster *k*-means solution. The median distance from the centroid of the Mesitas cluster to the points outside it (0.797) is lower than for either B97 or Fábrica San José (resulting from the large number of points that lie outside the principal cluster but not far from it), and thus indicates less productive differentiation than in either of these two communities.

A multidimensional scaling of household assemblages from Hongshan chiefdoms in the Western Liao Valley of northeastern China (4500–3000 BCE) shows similarities to both the diffuse Mesitas scaling (on one hand) and the more clustered scalings of Fábrica San José and B97 (on the other). The best-known Hongshan site is Niuheliang, where complexes of ceremonial architecture and elaborate burials with finely carved jades occur at more than 16 localities scattered through unproductive mountainous terrain; the distribution of associated occupation is as unusual as the architecture [31]. Niuheliang appears to have been a pilgrimage center for a vast region [32], and thus would likely have had an atypical local economy. A sample of 50 households from several dispersed Hongshan villages in the more productive Upper Daling Valley and nearby hills provides a more accurate view of regular economic life in Hongshan villages [33].

A multidimensional scaling of these 50 households (Fig 1D) shows a dense central cluster of similar artifact assemblages like the configurations examined previously. This cluster is considerably better defined than in the Mesitas configuration, but less clear than at B97 and much less obvious than at Fábrica San José. A six-cluster *k*-means solution identifies the red points in Fig 1D as its members. As at Mesitas, a sparser cloud of points extends fairly far beyond the cluster, but several outliers are strongly separated from it. The outliers and points at the fringes of the principal cluster are characterized by high proportions of axes, adzes, flake cores, blade cores, lithic shatter, retouched flakes, tools with especially obtuse or acute edge angles, projectile points, abraders, awls, drills, fine-edged choppers, and/or blades. In the Upper Daling configuration, the median distance from points outside the central cluster to the cluster centroid is 0.865, reflecting greater productive differentiation than at Mesitas but less than at B97, just as the intermediate characteristics of its scaling configuration would lead us to expect.

## Local economy, wealth, and political economy

A regional chiefly polity requires a political economy to fund the activities that integrate it and without which it could not exist. And the political economy itself must be funded by mobilizing resources of some kind [34,35]. In the context of a well-developed local economy, considerable household interdependence means that goods are moving between producers and consumers in substantial quantities, offering an excellent opportunity for redirecting some of them to fund a political economy, and for accumulation of wealth in the hands of elites. In the Western Liao Valley, this opportunity was sharply limited by the low level of productive differentiation and household interdependence, and these limitations were even more severe in the Alto Magdalena, with even lower levels of productive differentiation. With little opportunity to divert goods as they changed hands in a well-integrated local economy (i.e. one characterized by more than trivial productive differentiation and interdependence), political economies, when they emerged, were driven by religious belief and ritual activities, materialized in the construction of ceremonial facilities and tombs of prestigious, but not wealthy, individuals. As has been argued elsewhere [1,31,33,36], the very elaborate tombs in the Alto Magdalena and the Western Liao Valley are best interpreted as indications of strong prestige differentiation based on ritual and belief rather than of large accumulations of wealth in elite hands.

In Barinas and Oaxaca, on the other hand, the larger quantities of goods moving between producers and consumers does appear to have provided the opportunity for the accumulation of wealth by elites [1,18,37]. This shows up particularly well in the archaeological evidence for Oaxaca, where burials and residential architecture indicate a level of wealth differentiation not seen in the Alto Magdalena or the Western Liao Valley, including the possible centralized storage of goods [38]. Wealth differentiation is also indicated in analysis of household artifact assemblages from Fábrica San José that, unlike the analyses presented above, include artifacts indicative of wealth and well-being [1,21]; three households with productive specialties had artifact assemblages with elevated proportions of items indicative of wealth. Of much farther-reaching social implications, however, are the indications that differences in standards of living were pervasive, even in tiny villages and hamlets—precisely the pattern of differentiation that did not develop in the Alto Magdalena or the Western Liao Valley, even though these regions also had specialized elite-connected crafts in the form of monumental statues and jades carved into symbolic shapes [28,29,31].

Wealth differentiation, then, was developing through the Early and Middle Formative in the Valley of Oaxaca, and the operation of a political economy was in evidence by the beginning of the Middle Formative in the form of public buildings of modest size. During the latter part of the Middle Formative, the scale of public construction increased dramatically, and stone buildings clearly identifiable as temples appeared. By the end of the period one temple on a stone-faced platform adjacent to a public plaza had been destroyed and replaced by an elaborate, multi-room residential structure far larger than any that had existed before [20,39]. By this point it is clear that wealth accumulation and political economy had become inextricably intertwined. Ritual activity was certainly an important integrative force in the San José Mogote chiefdom, but the way it combined with strong wealth differentiation gave this chiefdom a very different shape than those of either the Alto Magdalena or the Western Liao Valley. This shape was a direct outgrowth of the way that stronger productive differentiation, with concomitant greater interdependence and exchange of goods (achieved early on in Oaxaca), provided opportunities for wealth accumulation that did not exist in the Alto Magdalena or the Western Liao Valley.

Differences in standard of living, while not as conspicuous as in Oaxaca, were also present in Barinas in the form of households with higher proportions of footed serving vessels, and larger houses on higher mounds [37], built to raise living spaces above the mud in this seasonally inundated region. As in Oaxaca, these differences emerged in the context of a local economy that was more highly integrated than those of the Alto Madgdalena or the Western Liao Valley. The elites of Barinas are also argued to have further enhanced the flow of goods between households by organizing the construction and cultivation of a 35-ha system of raised fields from which surplus was mobilized for a political economy that supported activities such as feasting and prestige goods exchange [23,24,40]. It also funded the construction of ceremonial platforms (up to 12 m high at the chiefly center of El Cedral) and palisades (some 2 km long on a raised earthen base at El Gaván). Wealth accumulation and political economy in Barinas, then, combined strongly not only with ritual as in Oaxaca, but also with defensive activities. Such combining of elements did not occur in the Alto Magdalena or the Western Liao Valley, where less integrated local economies led to political economies that were strongly ritual-focused.

## Expanding the comparison

It would be natural to expect that local economies with strong productive differentiation and economic interdependence would provide especially promising foundations for highly developed political economies. This seems to characterize the four cases discussed above, and well-integrated local economies also seem to characterize the development of political economies in early states (Valley of Oaxaca, Basin of Mexico, Titicaca Basin, Southern Mesopotamia, Yellow River Valley, Indus Valley, Nile Valley, Zimbabwe, etc.). But well-integrated local economies do not always pave the way to state-level organization, and attention to those that don’t can be enlightening. Toward this end, we now turn toward two such cases, ones widely held to have particularly strong productive differentiation.

Surprisingly early and elaborate ritual items have attracted much attention at Çatalhöyük on the broad, dry Anatolian Plain [41,42], but the local economy can also be explored at this settlement for Pre-Pottery Neolithic through Late Neolithic times (6500–4500 BCE). At this unusually large and densely packed settlement, artifact assemblages from a sample of 15 excavated houses provide a basis for evaluating productive differentiation [43]. A multidimensional scaling of these 15 assemblages (Fig 1E) has the by-now familiar central cluster of typical households with very similar artifact assemblages. This cluster, as in the Fábrica San José configuration, is very easy to define, but the gap between it and outlying households is even wider. The obvious pattern in Fig 1E is of course duplicated by a seven-cluster *k*-means solution, which identifies the red points in Fig 1E as the central cluster using the same systematic approach applied to the other scalings. The six outliers at the margins of the configuration represent assemblages with unusually high proportions of debitage, cores, hammerstones, choppers, anvils, abraders of several kinds, sanding slabs, polishing slabs, palettes, pigment, axe/celt preforms, querns, quern rough-outs, pestles, stone balls, shaft straighteners, hoes, mace heads, and/or weights. The median distance from the main cluster centroid to these outlying households is 1.334, reflecting the highest level of productive differentiation seen so far.

The Iron Age community of Jenné-Jeno, in the broad riverine zone of the Middle Niger Delta, is usually labeled “urban” at its peak (400–900 CE) [44–46], but Jenné-Jeno’s population of 7,300 is not much larger than the “village” of Çatalhöyük. Although not actually dating to the Neolithic period, Jenné-Jeno provides an especially useful point of comparison in this analysis, and does, in any event, pertain to the initial agricultural settlement of its region. Excavation units scattered across the site provide 31 domestic artifact assemblages from different locations within it [47]. A five-cluster *k*-means solution on the multidimensional scaling configuration (Fig 1F) identifies a principal cluster of 22 “ordinary” households, with the remaining nine households set off to varying degrees for high proportions of spindle whorls, ceramic and stone weights, and materials related to iron production. The median distance from the main cluster centroid to these outlying households is 1.473, reflecting greater productive differentiation than in any of the other archaeological examples discussed.

The well-integrated local economies of Jenné-Jeno and Çatalhöyük would seem to provide even better opportunities for wealth accumulation than in Oaxaca or Barinas, but the data for Jenné-Jeno and Çatalhöyük do not include any of the customary archaeological evidence for differences in wealth or standards of living, such as lavish burial offerings, especially spacious or comfortable residences for some families, or household assemblages with higher proportions of costly possessions [42,45,46,48,49]. In short, wealth accumulation, as well as other hierarchical forms of differentiation, are particularly weak in these social contexts—so much so as to generate (sometimes adamant) opposition to even calling them chiefdoms [45]. We do not disagree that these exceptionally large communities were remarkably non-hierarchical, nor do we find any utility in quibbling over what to call them. Indeed the fact that these societies do not fit some familiar typological stereotypes, or the patterns we have developed thus far, is precisely why there is much to be learned by including them in the comparison.

Jenné-Jeno and Çatalhöyük make it quite clear that, while a well-integrated local economy can provide an excellent opportunity for wealth accumulation, as we have seen for Oaxaca and Barinas, the emergence of wealth differences in such a situation is not automatic. Despite having especially well-integrated local economies, wealth accumulation did not occur at Jenné-Jeno or Çatalhöyük. Wealth differentiation, then, does not just happen whenever the opportunity presents itself—a well-integrated local economy invites wealth accumulation, but is not, by itself, a sufficient condition. This finding may call into question a notion that has been strong in a major part of the chiefdom literature: that self-aggrandizing behavior by ambitious individuals is ever present and, given the opportunity, will drive societal development [34,50–52]. Jenné-Jeno and Çatalhöyük did develop into local communities that were demographically much larger than any in Oaxaca, Barinas, the Alto Magdalena, or the Western Liao Valley, but wealth differentiation and other indicators of self-aggrandizement are absent at Jenné-Jeno and Çatalhöyük. Self-aggrandizing behavior could very plausibly be behind the wealth differentiation seen in Oaxaca and Barinas, or the ritual and prestige differentiation in the Alto Magdalena or the Western Liao, but aggrandizers are just not apparent in the archaeological record for Jenné-Jeno or Çatalhöyük. An alternative interpretation could be that ambitious individuals were present in these communities but the road to self-aggrandizement via the local economy was blocked. Organizational roadblocks could have included managing the challenges of a large settlement via segmentation rather than centralization or (what amounts to the other side of the same coin) the lack of mechanisms for community integration above the household scale, such as the ritual systems that were all-important in the Alto Magdalena or the Western Liao Valley, and that played important roles in Oaxaca and Barinas as well. Jenné-Jeno provides precious little evidence of ritual activity; and though such evidence is abundant at Çatalhöyük, it suggests that ritual operated at a resolutely household scale [53,54].

If aggrandizers are the drivers of political development, their absence, or obstacles to their action, could account for the negligible development of a political economy at Çatalhöyük, where the public products of a political economy are as absent from the archaeological record as are indications of wealth or prestige differentiation. Activities of aggrandizers seem equally absent from Jenné-Jeno, but here resources were mobilized in a political economy for the construction of a city wall that represented a substantial public investment [47]. This was by no means a record-setting level of investment in public works for chiefdoms, nor one that placed a particularly heavy per capita burden, or tax rate, on the population, but both the investment and the tax rate were higher than those of many other societies conveniently labeled chiefdoms [2,3]. The presence of a political economy of this magnitude in the seeming absence of aggrandizers would suggest that it was the result of bottom-up processes of self-organizing systems or collective action [18,46].

## Implications of productive differentiation and local economy integration

In sum, these comparisons illustrate that, as chiefdoms emerge, productive differentiation and local economic integration may develop very strongly or only very slightly, and that the degree to which local economies are integrated has implications for the shape that political economies take. A strongly integrated local economy opens a door through which aggrandizers can find ready opportunities, afforded by a vigorous flow of goods from producers to consumers, to accumulate personal wealth and to fund a political economy to further their political ambitions. This seems an accurate description of the central process by which political economies grew in Oaxaca and Barinas, but this is not how political economies developed in the Alto Magdalena and Western Liao Valley, which had only poorly integrated local economies. Political economies nonetheless developed in these regions, but the pathway their development followed led through a different door, one that did not depend on a well-integrated local economy. Differentiation was strongest in the realm of ritual and prestige rather than wealth. Resources for the political economy were not siphoned from the goods moving through a local economy, but were mobilized directly (probably in the form of labor) for the creation of ritual facilities and the enhancement of individual prestige.

While we have described these as two qualitatively different pathways, it does not amount to a simple dichotomy—it is a question of the relative importance of action in different societal spheres. In minimizing the degree of wealth accumulation in the Alto Magdalena and the Western Liao, we do not mean to imply that everyone was exactly equal in terms of standard of living, only that such differences were minimal. Wealth differences are clearer in the archaeological record of Oaxaca and Barinas, but ritual and prestige differentiation were still stronger than wealth differentiation was in the Alto Magdalena and the Western Liao. In Oaxaca and Barinas, the political economy involved a broader combination of ritual and prestige with wealth and productive differentiation, whereas in the Alto Magdalena and the Western Liao political economy was more narrowly focused on ritual and prestige. What happened in these regions is thus consistent with previous arguments [34] that resources in a political economy centered on personal wealth are easily diverted to ritual purposes and prestige enhancement, while resources mobilized directly for the creation of ritual facilities or the enhancement of prestige are not easily converted into personal wealth.

Much of this variation in the political economies of these four chiefdoms, then, would seem to track back to variation in the extent to which productive differentiation developed in local economies. More integrated local economies in Oaxaca and Barinas opened a door that was closed to aggrandizers in the Alto Magdalena and the Western Liao Valley. This same door would have been opened especially wide by the extremely well-integrated local economies of Jenné-Jeno and Çatalhöyük, but as these cases demonstrate, just because a developmental opportunity opens up does not necessarily mean it will be pursued. Why not is a very interesting question, but pursuing it would take us far from our objectives here. It is nonetheless worth considering whether the intensification of productive differentiation and well-integrated local economies of Jenné-Jeno and Çatalhöyük owe something to the absence of aggrandizers siphoning off wealth or to the low labor burdens their minimal political economies placed on their populations.

Variation in local economies thus played a vital role in giving these six chiefdom societies very different shapes. This automatically leads to the question of how these local economies came to develop so differently in the first place. What we have characterized as well-integrated local economies involve high levels of interaction in the form of the exchange transactions that move goods from producers to consumers; in less well-integrated local economies there is correspondingly less interaction of this kind. The spatial implications of interaction are well known and have long been formulated in distance-interaction principles, which are thus a promising avenue of exploration for understanding how local economies develop.

## Distance and interaction

The village has always loomed large in archaeologists’ imaginations as the social context for household interaction in early complex societies [55–58]. The large aggregated populations of villages facilitated the pooling of labor for communal tasks in the realms of subsistence, defense, or ritual; fostered social institutions that provided for effective information transfer and conflict resolution; and, of most relevance here, encouraged the integration of local economies. This same importance of interaction in aggregated populations has long been recognized at the scale of cities. It has been such a common perception, in fact, that it comes as no surprise to read Paul Krugamn in the New York Times saying that cities “offer large markets, ready availability of specialized suppliers, large pools of workers with specialized skills, and the invisible exchange of information that comes from face-to-face contact” [59]—all of which amount to different forms of interaction. The surprise comes in noticing that this characterization of cities is virtually identical to what archaeologists have long said about Neolithic villages as a fertile seedbed for developing local economies. We are all certainly accustomed to thinking about cities and villages as very different phenomena, but a growing chorus of studies argues that processes related to aggregation in both modern and ancient urban systems operated in the same way in the village-centered systems of archaeologically known chiefdoms [15,16,60–62].

Research on modern cities has strongly emphasized the factors enumerated by Krugman above, which comprise the fundamental economic dynamic of urban aggregation [63–66]. This dynamic creates the spirals of urban growth seen on all continents today by way of the mutual feedback between population aggregation and economic growth: as populations aggregate, economic growth is promoted, and this further increases the centripetal forces that draw people to cities. Archaeologists have not paid as much attention as we might to the way these processes of aggregation and economic growth operated in early villages, where the mutual feedback and spirals of growth observed in modern cities ultimately had their beginnings. These beginnings lie especially in the exchange interactions that we have discussed above for well-integrated local economies.

Distance-interaction principles, if often not well understood, are nonetheless a hoary staple of many social science disciplines [67–73]. The basic idea is a simple one: without modern communication or transportation technology, people interact more frequently with others at close range than at longer distances [74]. This higher frequency of interaction at shorter distances is a question of averages. It does not preclude long distance interaction (which we regularly see evidence of in the archaeological record); it means only that there is a general tendency for interaction frequency to decrease as distance increases. Archaeologists have often accepted this idea (at least implicitly) for interaction at regional and inter-regional scales, but the principle applies at very local scales as well.

The relationship between distance and interaction at this local scale is not an abstract law of nature, but simply an empirical generalization easily observed in our own daily lives. For example, without modern technology, the production and distribution of goods that circulate in a local economy account for a large proportion of local interactions. Perishable food like bread is the epitome of such a good, whether the bread is tortillas, pitas, or baguettes. Even in modern times, bakeries are found at relatively short intervals (at least where processed bread in plastic bags is not desired). Tourist guidebooks have exclaimed that Paris, for instance, seems to have a *boulangerie* every 100 m. This was only a slight exaggeration in 1950, when the actual distance was, on average, about 121 m. Some approximate work with the total number of bakeries in France suggests that this has increased to 158 m at present [75]. Bread buying in this context is a virtually daily activity, and this high frequency is clearly reflected in the close proximity of bakeries to their customers. Drugstore purchases are also made frequently, but not as frequently as bread purchases, and drugstores occur at an average interval of 188 m in Paris. Trips to buy wine may be especially frequent in Paris, but typically less frequent than drugstore purchases; as a consequence, wine stores occur at an average interval of 230 m. Parish churches, visited still less frequently by most people, are 498 m apart on average [76–78].

Even in a modern city, then, with an excellent public transportation system, fundamental distance-interaction principles operate strongly at a very local scale—higher frequencies of interaction correspond to shorter distances of travel. Without cars, buses, or telephones—let alone online shopping—the impact of distance on interaction would have been even heavier in Neolithic villages. These villages often placed hundreds of inhabitants within a few hundred meters of each other for the first time, creating opportunities for more intensive local interaction than had ever existed before. We do know that such villages occurred occasionally in non-agricultural contexts, but most often subsistence was based on farming. Substantial reliance on plant cultivation, though, would make it attractive for households to live, not near other households, but directly on the plots that they cultivate, so as to minimize the time they spend traveling to their fields [79]. This is also a consequence of distance-interaction principles, but one in which the distance and the interaction are not between people but between farmers and the land that they cultivate. In deciding where to live, then, households must balance competing forces that pull them toward their fields (on one hand) and toward other households (on the other). Dispersed farmstead dwelling makes cultivation less laborious by eliminating walking to fields, although interaction with other people is more costly because they are so spread out. Village dwelling increases the investment in walking to fields but facilitates all forms of interaction with other people. These forms could include cooperation in agricultural labor, ritual activities, purely social encounters, and, most important for present purposes, the transactions of a local economy. Early complex societies balanced these competing forces in different ways, resulting in considerable variation in household spacing.

## Geometry of household spacing

### Frequency of interaction

**Fig 2:**
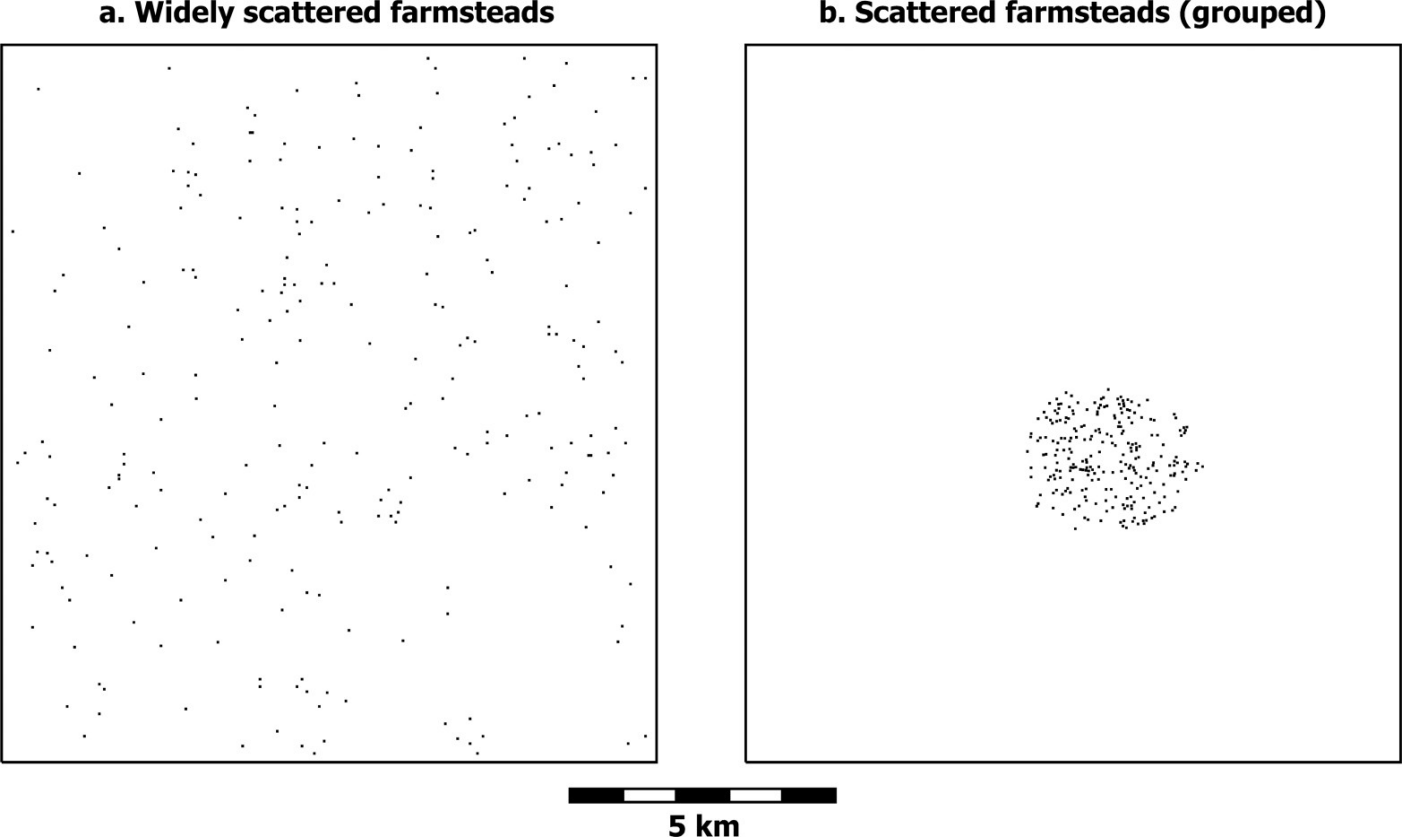
Two hypothetical farmstead distributions. a) widely scattered farmsteads and b) farmsteads grouped more closely together within the same area. Average nearest neighbor distance is 440 m in Fig 2A and 88 m in Fig 2B. Both images have 250 households and thus the same regional population density (7.5 persons/km^2^). Based on modern household distributions in southwestern Colombia.

Regional population densities at the beginning of the Neolithic were usually quite low, often on the order of 5–10 persons per km^2^ or even less. At such low regional densities, if farmsteads spread themselves throughout a large territory, neighbors are necessarily very far apart—over 400 m on average to the nearest neighbor in the example in Fig 2A. (This and the following examples of household spacing are hypothetical; they were invented to provide clear illustrations of abstract principles but with a basis in reality, as discussed in *Material and Methods*.) This means that having a word with your closest neighbor would mean a round-trip walk of some 10 minutes on average (at a pace of 5 km/hr). From a farmstead in the center of Fig 2A, a walk to a more distant neighbor would be 2 hours (the average round-trip travel time to all the other 249 farmsteads on the map). Higher regional population densities, of course, would push farmsteads closer together and reduce the travel costs of interaction, but overall regional densities high enough to make much difference in this regard did not occur until well after the beginning of the Neolithic.

Even a small regional population in the early Neolithic, however, could locate farmsteads relatively close to each other in one part of a region, thus mimicking the effect of high regional density and facilitating interaction with neighbors. In Fig 2B, the overall regional population density is the same as in Fig 2A (7.5 persons/km^2^), but a density of 160 persons/km^2^ has been created among the households by grouping them more tightly together in the center of the map. Here a household in the center of the group would have an average round-trip walking time of around 25 minutes to the other 249 households, less than one-fourth as long as in the more dispersed farmsteads in Fig 2A. Just how closely farmsteads can be spaced depends, of course, on the amount of land required to support a household. This limit will vary from region to region, but it can be transcended by separating farmland from living space, allowing households to be spaced closer together in what is, in effect, a village. The group of farmsteads in Fig 2B cannot comfortably be labeled a village because, at 3.5 km across, its households are too widely scattered to permit the level of daily interaction between households that has long been used as a criterion of village life [80]. The closer household spacing within villages facilitates more frequent interaction with neighbors, although a penalty must be paid in the form of walking longer distances to cultivate fields, which are no longer directly adjacent to houses.

**Fig 3:**
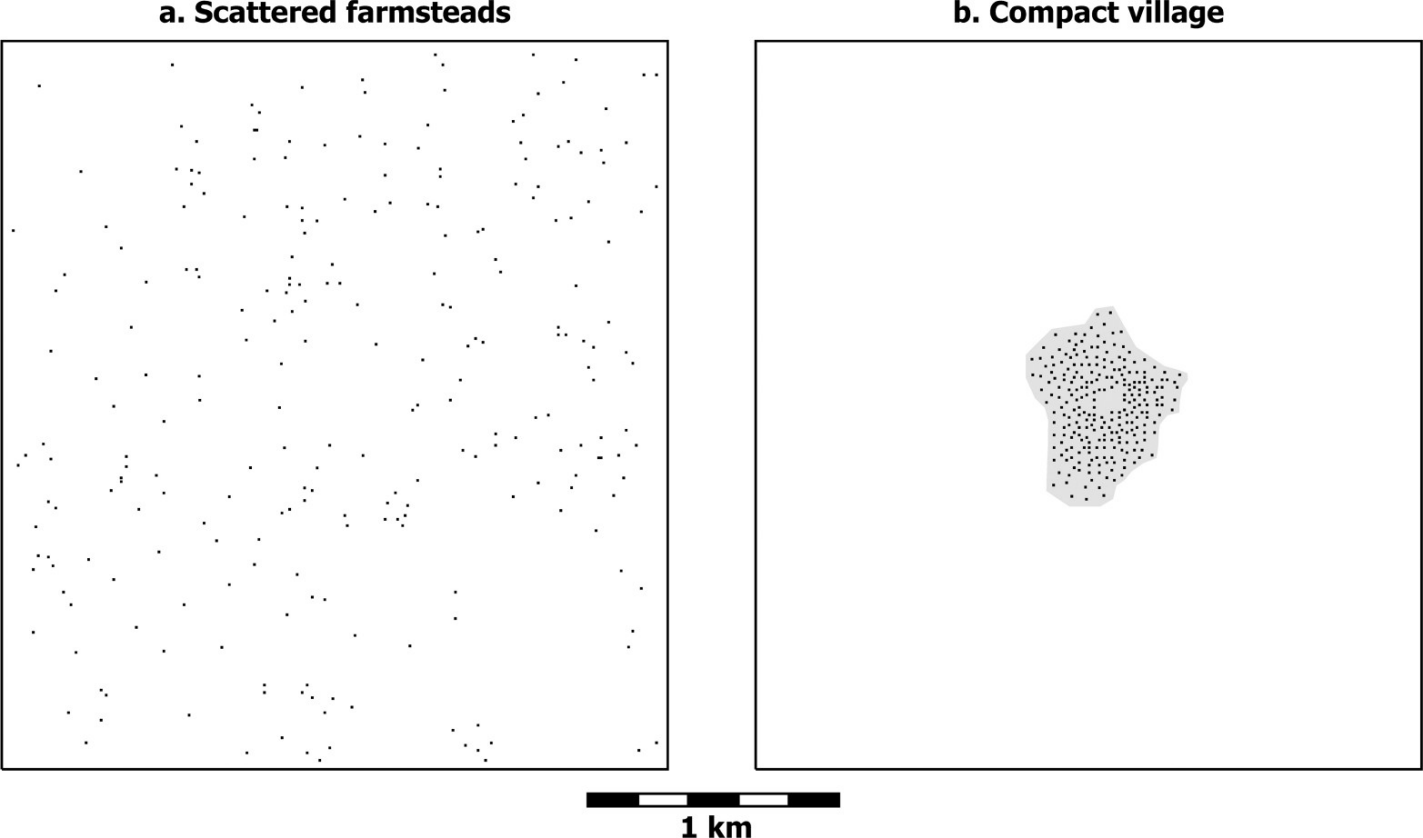
Hypothetical farmstead and compact village distributions. a) scattered farmsteads and b) a compact village (shown at a larger scale than Fig 2). Average nearest neighbor distance is 88 m in Fig 3A (the same as in Fig 2B) and 13 m in Fig 3B. Gray hatch in Fig 3B corresponds to the village area (30.7 ha). Both images have 250 households. Based on modern household distributions in southwestern Colombia.

It is enlightening to continue to work through the math of these simple geometric principles as household spacing varies in a series of other example distributions consisting of 250 households each. All these examples are slightly modified versions of actual modern household distributions and thus reflect some of the oddities of the real world. Fig 3A shows a settlement pattern consisting of 250 dispersed farmsteads at an overall density as high as that within the group in Fig 2B. A household that interacted with all the other 249 households would walk an average of 25 minutes to reach any of these neighbors and return (the same as in Fig 2B since the distances to neighboring households are a function of density, and the densities in the two examples are the same). On the other hand, for a household in the compact village of Fig 3B, the average round trip to all 249 other households would be only 5 minutes. Though neither a 5 minute walk nor a 25 minute walk seems much of a burden, we do react differently when asked to walk 25 minutes in contrast to 5. (The latte consumption of one of us increased radically when a coffee shop opened just down the block, even though lattes had previously been available for less than a half-hour round-trip walk.)

Travel time required for farmers to cultivate their fields turns out to be almost exactly the inverse of travel time for interaction with neighbors. A farmstead dweller in Fig 3A accepts a 25-minute walk to neighbors in exchange for an average round trip of only 2 minutes to cultivate any spot in the adjacent fields. A village dweller in Fig 3B reaches any neighbor and returns in no more than 5 minutes, but at the cost of an average round trip of 26 minutes to fields surrounding the village. (These calculations assume, for comparability, that the frames of Fig 3A and 3B delimit the farmland that supported the 250 households; the only difference is how those households distributed themselves within that territory).

For early farmers, then, the choice between compact village and scattered-farmstead living was a pretty even trade-off. Villagers had very accessible neighbors with whom interaction was easy, but this came at the cost of longer walks to their fields; farming plots were as accessible to farmstead dwellers as neighbors were to villagers, but interaction with very many neighboring farmsteads involved walks as long as those villagers took to their fields—or even longer if farmsteads were not fairly close together (as they are in Figs 2B and 3A). As settled farming life got underway, these farmers would thus seem to have had a stark choice to make: easy interaction with neighbors or easy access to their fields.

**Fig 4:**
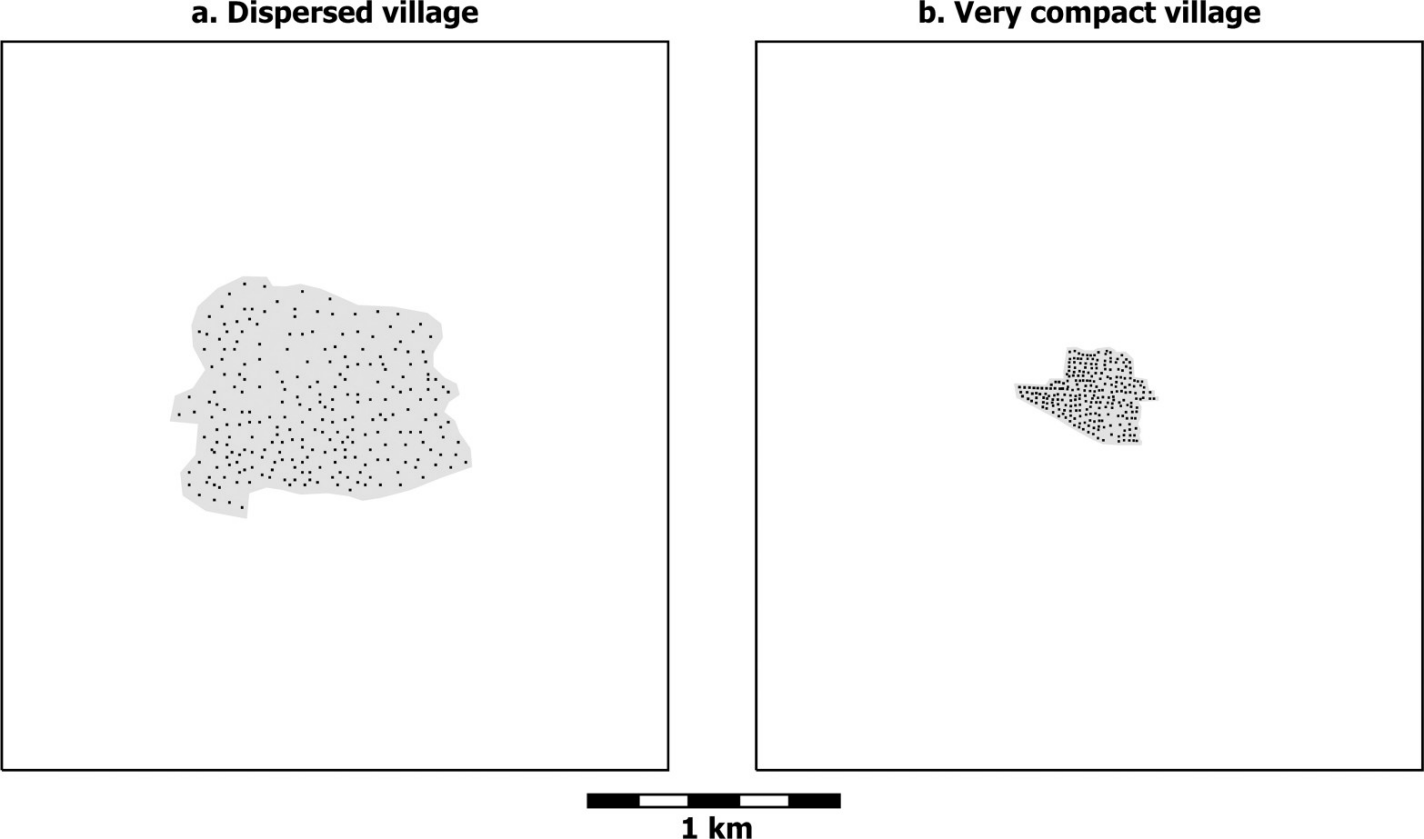
Hypothetical dispersed and very compact village distributions. a) a dispersed village and b) a very compact village. Average nearest neighbor distance is 21 m in Fig 4A and 10 m in Fig 4B. Gray hatch corresponds to the village area (79.5 and 12.1 ha, respectively). Both images have 250 households. Based on modern household distributions in a) southern Tanzania and b) Khuzestan, southwestern Iran.

**Fig 5:**
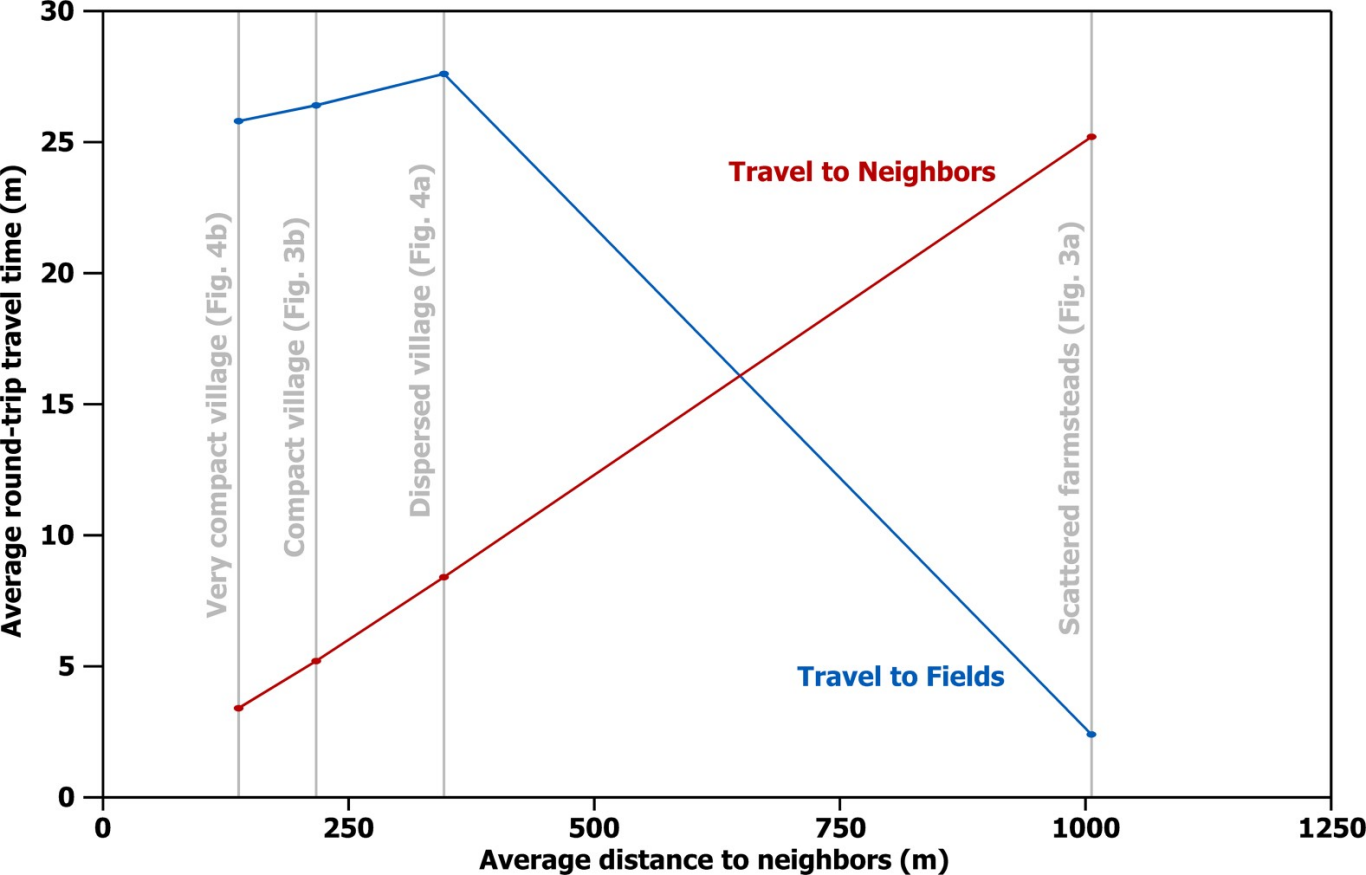
Walking times. Average round-trip walking time to cultivated fields (blue) and neighbors (red) for each of the four household distributions in Figs 3 and 4 (gray).

But this choice is not as simple and binary as it seems at first. Making a dichotomy between villages and scattered farmsteads is conceptually easy (this is why dichotomies are always tempting), but, as is often the case, this dichotomy fails to do full justice to reality. The spacing between scattered farmsteads is not a universal constant, but is closer when regional population densities are high or farmsteads are grouped in only part of a region (as they might do to take advantage of a particularly favorable environmental zone, for instance). The limit to how close farmsteads can be to each other depends on the amount of land they need to sustain themselves, which varies with the richness and spatial distribution of a region’s agricultural resources. Likewise, not all villages were created equal, but vary considerably in terms of internal household spacing. Household spacing is therefore not a binary variable (farmsteads vs. village) but is measured most meaningfully along a continuous scale. At the low end of this scale are very tightly packed villages (e.g. Fig 4B). From there spacing increases through still compact but less tightly packed villages (e.g. Fig 3B) and on through more dispersed villages (e.g. Fig 4A). At least some farming is usually practiced within dispersed villages, often in the form of infield-outfield cultivation, and cultivation even sometimes occurs in compact villages in the form of small gardens [81,82]. Villages still more dispersed than that in Fig 4A—with even greater average household spacing—are imaginable, where some households practice infield-outfield cultivation and others have larger plots and can sustain themselves by cultivating the land immediately around their houses. Eventually, as spacing continues to increase, dispersed villages become so spread out that interaction between households becomes highly attenuated, and they grade off into what is more accurately described as scattered farmsteads (e.g. Fig 3A). In this way, the scale becomes a continuous one ranging from villages with extremely tightly packed houses to very loosely scattered farmsteads. The graph in Fig 5 illustrates how travel times to neighbors and travel times to fields behave inversely as household spacing changes.

### Interaction network size and composition

**Fig 6:**
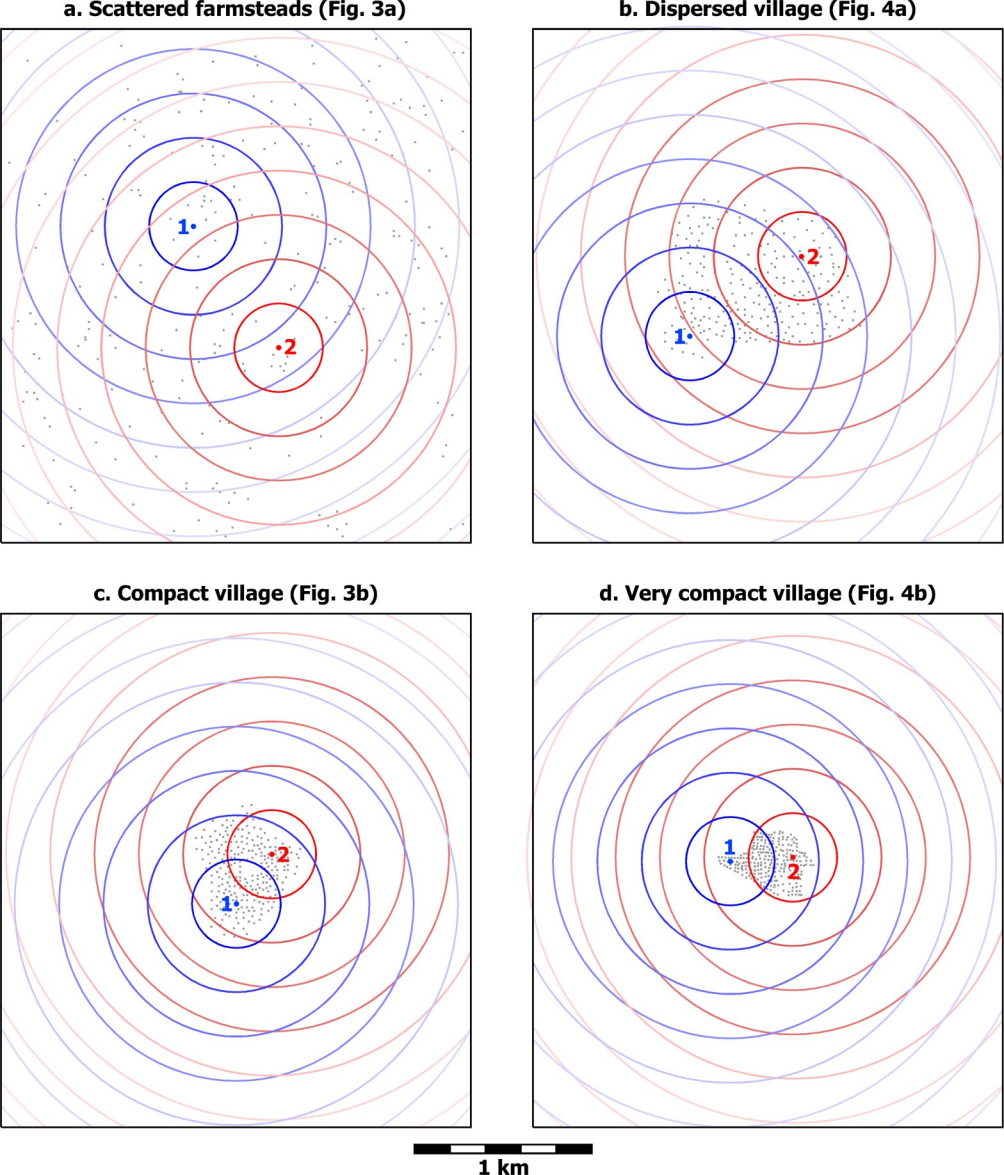
Interaction networks. Overlapping interaction networks of two households in each of the four household distributions in Figs 3 and 4. Fading colors reflect the diminishing average frequency of interaction at intervals of 250 m.

A further implication of the geometry of household spacing is that the number of neighbors a household has (that is, the size of its interaction network) increases with distance. Among farmsteads scattered across a large area, distance-interaction principles indicate that no two households would have exactly the same interaction network. This general expectation is represented by rings of increasing size around households 1 and 2 in Fig 6A. As the rings get larger, more and more neighbors are included in each household’s network, but these larger networks come only at the expense of decreasing average intensity of interaction, signified by the fainter and fainter colors of the larger blue and red circles.

**Fig 7:**
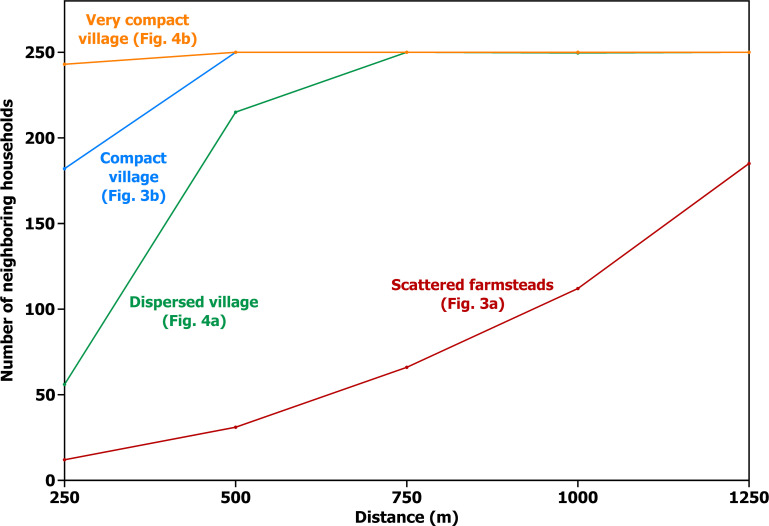
Numbers of neighbors. Neighbors within a series of interaction networks (at 250 m intervals) for each of the four household distributions in Fig 6.

Fig 7 shows how the number of neighbors increases with distance for a farmstead in Fig 6A, slowly at first, but with each larger circle, the area, and thus the number of neighbors, increase more rapidly. If the scattered farmstead distribution continued at a similar density beyond the artificial boundaries of Fig 6A, the number of potential neighbors would continue to increase in this way until the natural limits of the region were reached. At such distances, or even at the larger distances included within the frame of Fig 6A, the average intensity of interaction would be greatly diminished. The two most distant neighbors within that map, for example, are almost 4 km apart, representing a round-trip walking time of over 1.5 hours. At this distance, daily interaction with many neighbors is just not possible. There would be no fixed limits to interaction, but it would gradually get harder and harder, and thus less and less frequent on average.

For the dispersed village in Fig 6B, the tighter packing of houses means that the number of neighbors increases much more rapidly with distance at fairly close range (Fig 7). Unlike the theoretically limitless farmstead distribution in Fig 6A, however, the increase in number of neighbors comes to a halt at the spatial limits of the village, which introduces a boundedness absent from scattered farmstead distributions. This boundary divides residential space from unoccupied farming space; the example in Fig 6B includes enough space around houses for infield cultivation within the village, but outfields beyond the boundary would likely be required to sustain a population of 250 households. The average round trip to visit a neighbor within this boundary is approximately 8 minutes, making interaction among all households on a virtually daily basis highly practical. There is, then, a social entity that does not exist in Fig 6A—one that would be called a local village community by the traditional criterion of mutual interaction on a daily basis.

Fig 7 shows that, as expected, the compact and very compact villages in Fig 6C and 6D position the 250 households within even shorter walking distances of each other, but the average round-trip travel time to neighbors does not decrease very meaningfully—from about 8 to 5 to 3 minutes for the dispersed village (Fig 6B), the compact village (Fig 6C), and the very compact village (Fig 6D), respectively.

**Fig 8:**
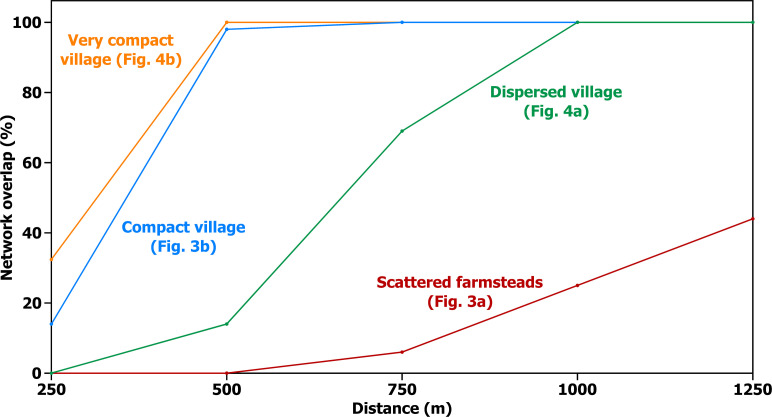
Network overlap. Overlap of interaction networks of Houses 1 and 2 (Fig 6) at varying distances (expressed as a proportion of total population).

Fig 6A also shows how the interaction patterns of two farmsteads would overlap, involving relatively frequent interaction with some of the same neighbors, although close neighbors of one household are only more distant neighbors for the other. There is no overlap between the closest neighbors of households 1 and 2; but beyond 500 m the concentric rings do overlap, and the number of shared interaction neighbors continues to increase with distance (Fig 8). This increasing overlap, though, comes at the cost of increasingly attenuated interaction between farmsteads because considerable distances are required to create it. This cost is much reduced in all three village examples, which not only bring all 250 households within about 1000 m of each other—a practical distance for maintaining frequent interaction—but also create plausible daily interaction networks that are identical for every household in the village. This is a direct outcome of the boundedness of villages noted above, and reinforces the way this boundedness delineates local communities as social entities of a kind that the geometry of scattered farmsteads does not create. Such a social entity provides favorable circumstances not just for cooperation, but especially for cooperation that—beyond benefiting households individually—benefits a defined group collectively [83].

The previous examples explored how travel times to neighbors and fields, numbers of close neighbors, and overlap of close interaction networks vary with household spacing while holding the total number of households constant at 250. Relaxing this constraint on total number of households leads to a final observation on the geometry of household spacing and the character of local interaction networks. If we take 1000 m (a 25-minute round trip) as a rough practical limit to interaction on a daily basis, the range of household spacing we have considered has dramatic implications for the overall size of the local network. We can use the average spacing from the four examples in Fig 6 to explore this issue. For farmsteads spaced like those in Fig 6A, there would be only 25 households within this practical limit (that is, within a circle 1000 m across). This number would increase to about 250 for the dispersed village in Fig 6B, the same total as in the example, since the village, though irregular, covers about the same area as a circle 1000 m in diameter. For the compact and the very compact villages, a circle 1000 m in diameter represents a substantial enlargement of the area covered by each village. If this larger area were occupied by houses at the same spacing as in the original examples, there would be 640 households in the compact village, and over 1600 in the very compact village.

With respect to the average time required to visit neighbors in a set of 250 households (or to reach agricultural fields), scattered farmsteads differ substantially from villages, but there is not much difference between the dispersed, compact, and very compact villages (Fig 5). The compact and very compact villages are also similar with respect to the proportion of network overlap, though here the dispersed village pulls out into a position intermediate between the compact village and scattered farmsteads (Fig 8). The dispersed, compact, and very compact villages also seem similar with respect to the numbers of neighbors they afford at different distances (Fig 7), but this impression is entirely a result of holding the number of households constant at 250 through all four examples. If this limit is removed and what is held constant is the approximate distance at which daily interaction is practical, a dramatic difference emerges between the villages—not only between the dispersed village and the others but also between the compact and very compact villages. Tighter packing of households in increasingly compact villages provides ever larger numbers of households in close interaction, and this has implications for the kinds of activities that generate interaction.

This is implicit in the spacing of foci of different kinds of interaction in Paris, as discussed above. *Boulangeries* are closest, not only because they are visited most frequently, but also because a relatively small-scale bakery can be sustained by the modest number of customers residing within a short distance. Drugstores and wine stores are spaced farther apart, not only because they are visited less frequently, but also because they need a larger number of customers to be profitable. By the same token, a parish church would call for an even larger demographic catchment (not least because less than half the population of France is Catholic, and even fewer actually practice). Paris is, of course, a vast demographic phenomenon, but the same interaction principles apply at the much smaller demographic scale of Neolithic villages.

## The archaeology of household spacing

Average household spacing is easily imagined in abstract examples and directly observed in the contemporary world. It is almost never possible, however, to actually measure the distances between all the houses in an archaeologically excavated village and average them to determine average household spacing. A more practical approach is to use the estimated population and the area of a village to obtain the *residential density* within that village. This is, in fact, the way in which the average household spacings in Figs 3 and 4 were determined.

Taking five persons as the average number of people in a nuclear-family household, the scattered farmsteads in Fig 3A represent a residential density of 1.6 persons/ha. Since this example of scattered farmsteads is unbounded, the residential density is equivalent to the regional population density, although regional density would usually be expressed in terms of persons/km^2^. The dispersed village in Fig 4A has the same number of households (250) in the same region (the map frame enclosing 7.685 km^2^), so the regional population density remains the same as in Fig 3A, but the area of the village within which the households are located is 79.5 ha, yielding a residential density for the dispersed village of 15.7 persons/ha. The compact village in Fig 3B has a residential density of 40.7 persons/ha; and the very compact village in Fig 4B, a residential density of 103.3 persons/ha.

Like the examples of abstract geometry above, the six archaeological cases whose local economies were examined show substantial variation in terms of residential density. The lowest residential density, 1.4 persons/ha, comes from Mesitas in the Alto Magdalena, where the 75 Regional Classic households used in the assessment of productive differentiation were scattered across a study area of 2.7 km^2^ [30]—almost exactly the same as the density of scattered farmsteads in Fig 3A. Although the residential density at Mesitas was somewhat higher than in other parts of the Alto Magdalena, it represents a household spacing that would still have allowed families to sustain themselves by farming the land immediately adjacent to their houses. The Alto Magdalena, then, is an archaeological example of scattered farmsteads in which distinct villages or local communities did not exist [29,84].

Hongshan villages in the Western Liao Valley are recognizable, although their boundaries are diffuse [85,86]. At a residential density of some 15 persons/ha [87], they were quite similar to the abstract example of dispersed villages in Fig 4A. (Much higher residential densities have been calculated for excavated portions of Hongshan villages, but the lower estimate is based on regional survey data, and avoids the bias that the most densely occupied portions of archaeological sites are the ones consistently chosen for excavation.)

Villages in the Barinas region in the western llanos of Venezuela were more clearly defined than in the Western Liao Valley and separated by considerable distances of open landscape. Residential densities within these villages, averaging some 25 persons/ha in Late Gaván times [40], were higher than the dispersed village example in Fig 4A but lower than the compact village in Fig 3B. In the Valley of Oaxaca, clearly bounded local communities were where most people resided, and the cultivated landscape around them had only the occasional small hamlet. Residential densities within these villages were similar to those in the compact village example (Fig 3B), beginning around 20 persons/ha in the first villages and climbing to 35–40 persons/ha or even more for at least some communities in the ensuing centuries [88,89].

Residential densities as high as that of the very compact village example in Fig 4B are rare in early complex societies, but did occur in some. Iron Age Jenné-Jeno in the Middle Niger Delta had a population estimated at 7,300 and a residential density of about 220 persons/ha [44], double the density of the very compact village example. An even more extreme example is found at Çatalhöyük in Anatolia, where, at the end of Pre-Pottery Neolithic and through Late Neolithic times, residential density may have been over 400 persons/ha for the East Mound, with a total population of nearly 6,000 [90]. Here family residences were not separate structures but joined together to form an extremely large apartment block. That these communities have both the largest populations and the highest residential densities of the settlements in our comparison corresponds broadly to the observation made by Ortman et al. [62] that, across an enormous range of space and time, larger settlements tend to be more tightly packed than smaller ones. The varied residential densities of communities that are similar in population size in the other regions we have considered, however, amount to variation from the broad averages that produce Ortman et al.’s power law—variation whose investigation can be enlightening, just as Ortman et al. suggest.

## Household spacing and productive differentiation: A functional relationship

Mesitas, Upper Daling, B97, Fábrica San José, Çatalhöyük, and Jenné-Jeno span practically the entire spectrum of household spacing, from widely scattered farmsteads to extremely nucleated villages. They also span a wide spectrum of variation in the integration of local economies, making it possible to explore the relationship between these two variables beyond the farmstead/village dichotomy. The two variables turn out to be very strongly correlated, with productive differentiation, and thus integration of the local economy, increasing as household spacing decreases (Table 1). There are, of course, only six cases in the analysis, but the rank order correlation (*r*_*s*_ = 0.943) between residential density (household spacing) and the median distance of outliers from the main cluster centroid in artifact assemblage scalings (productive differentiation) has very high statistical significance (*p* = 0.005). This correlation is exactly what the geometry of household spacing discussed above would lead us to expect, because (1) greater productive differentiation entails greater economic interdependence in a more integrated local economy, (2) interdependence requires exchanges of goods in economic interaction, and (3) closer household spacing facilitates any kind of interaction. The correlation between household spacing and productive differentiation thus reveals a functional relationship for these six cases. Similar observations have been made elsewhere on the basis of both plausibility and archaeological analysis [1,74,91–93].

**Table 1:** Measures of productive differentiation and residential density.

Community	Productive Differentiation Index	Median Distance between Outliers	Residential Density (persons/ha)
Mesitas	0.797	1.15	1.4
Upper Daling	0.865	1.40	15.0
B97	1.088	1.71	25.0
Fábrica San José	1.187	1.98	35.0
Çatalhöyük	1.334	2.42	422.0
Jenné-Jeno	1.473	2.01	221.0

The scaling configurations provide additional clues about how productive differentiation took shape in early chiefdom communities. In the configurations where there are distinct outliers, representing households with particular emphasis on certain productive activities, these outliers are sharply separated not only from the main cluster but also from each other. Multiple households characterized by the same unusual set of activities would be close to each other in the configuration and appear as a tiny outlying cluster (as opposed to a single outlying point), but this pattern does not occur in any of the configurations. Instead, as outliers become stronger in the progression from scattered farmsteads to very compact villages, they are not only more and more distant from the main cluster but also more widely separated from each other. This is evident in the median distance between outlying points in each configuration (that is, those not included in the main cluster), which increases from 1.15 for Mesitas, to 1.40 for Upper Daling, 1.71 for B97, 1.98 for Fábrica San José, 2.01 for Jenné-Jeno, and 2.42 for Çatalhöyük (Table 1). The correlation between these values and overall strength of productive differentiation (as measured above) is not perfect but very strong and significant (*r*_*s*_ = 0.943, *p* = 0.005).

In these communities, then, the particular combinations of activities that differentiate some households strongly from the main cluster are unique to each household and not repeated in others. These data do not show the emergence of standardized productive specializations that recur consistently in more than a single household. In other words, these communities do not seem to have “butchers, bakers, and candlestick makers”, or any other well-defined specializations regularly pursued by multiple households. Such a pattern would be consistent with growing but still modest emphasis on particular productive activities in food-producing households that were just beginning to supply some kinds of goods to their neighbors. This is clearly the case for Mesitas, Upper Daling, Fábrica San José, B97, and Çatalhöyük, where the degree of productive differentiation does not even rise to the threshold of some definitions of “craft specialization.” Iron production at Jenné-Jeno comes closer to a standardized productive specialization than anything we see in the other cases, but even here the assemblages reflecting iron production are surprisingly varied.

These societies thus show productive differentiation without the standardized specializations that archaeologists often imagine for complex societies [94]. But even at these early stages, the degree to which the local economy was integrated varied substantially, and this variation correlates strongly to household spacing within local communities.

## Conclusions

Although the local economy has received relatively little archaeological attention (compared to long-distance exchange or the production and distribution of luxury goods for export), we have seen that its degree of integration is an important variable in the half-dozen early chiefdoms we have looked at. The local economy, after all, comprises the bulk of economic activity in most early complex societies, and is thus fundamental to the dynamics of social change. For this reason, it is useful to understand what leads to variation in the integration of the local economy, which is to say in the degree of productive differentiation and interdependence. Since productive differentiation and interdependence revolve around household interaction, and since household interaction is facilitated by closer household spacing, it is not surprising that more strongly nucleated settlements lead to more strongly integrated local economies.

In Oaxaca and Barinas, compact villages facilitated well-integrated local economies that offered ready opportunities for funding political economies and for wealth accumulation, though ritual activities also played an important role. In contrast, more attenuated interaction in the dispersed villages of the Western Liao Valley and among the scattered farmsteads of the Alto Magdalena impeded the integration of local economies, limiting their potential as a source for funding political economies. Effectively lacking that source, ritual activity became much more central to sociopolitical integration in these regions. At Jenné-Jeno and Çatalhöyük, extremely high residential densities and very large populations correlated, as expected, with very high levels of productive differentiation in the local economy. This would presumably have created even greater opportunities for wealth accumulation than the local economies of Oaxaca and Barinas, but there is no evidence for substantial wealth differentiation at Jenné-Jeno or Çatalhöyük. Also absent from these cases is the evidence for supra-household ritual that was so important to sociopolitical integration in the Alto Magdalena, the Western Liao Valley, Oaxaca, and Barinas.

In the six early chiefdoms dealt with here, variation in settlement nucleation led to exactly what we would expect in the integration of local economies based on distance-interaction principles. And this, in turn, led to differences in the shape that political integration took in Oaxaca, Barinas, the Alto Magdalena, and the Western Liao Valley. The observations we have made above about political integration in these regions relate to what is often discussed as different bases of political power [34,95]. The relative importance of different bases of power has often been cited as a central component in early complex society variation, but what led to these differences in the first place has not received much attention. A focus on interaction, settlement nucleation, and local economy integration, however, has shown us how one pathway that starts from strong settlement nucleation leads to an emphasis on economic bases of power (complemented by ritual) in Oaxaca and Barinas, and how a different one that starts from greater settlement dispersal leads to an emphasis on religious bases of power in the Alto Magdalena and the Western Liao Valley. Broader comparison of more regional trajectories of chiefdom development is beginning to suggest that these two pathways are recurring patterns in early complex society variation.

These are, however, by no means the only two patterns to be seen. Jenné-Jeno and Çatalhöyük show even stronger settlement nucleation and local economy integration than even Oaxaca, but these features did not lead to well-funded political economies, accumulation of wealth, or strong economic power (or strong power of any kind). Accounting for the lack of powerful individuals at Jenné-Jeno and Çatalhöyük, despite their large demographic scale, has been a long-standing intractable problem. Focusing on settlement nucleation and local economy integration has not solved this problem, but it has made it clear that in both cases a well-integrated local economy provided an ideal opportunity for wealth accumulation and economic power. Efforts to solve this problem might most productively focus on what factors prevented this opportunity from being taken advantage of. Some possibilities for such factors were suggested in the discussion above.

We have just discussed the relationship between settlement nucleation and local economy in terms that make settlement nucleation the “cause” and local economy integration the “result.” For Oaxaca, Barinas, the Western Liao Valley, and the Alto Magdalena, we can see that this is indeed an accurate account of the direction of causality. Patterns of household spacing were established at the shift to sedentary agricultural living, and it was these patterns that subsequently led to more integrated or less integrated local economies. The functional relationship between household spacing and local economy, however, is equally consistent with causality in the reverse direction: nascent productive differentiation could encourage more nucleated settlement, since the local economy could itself become a centripetal force drawing households together into more compact settlements. Probably of greatest consequence, nucleation and the local economy can develop into a relationship of mutual causality, creating a growth spiral in which the benefits of a well-integrated local economy draw more people into a settlement, spurring even greater economic integration, which further strengthens the centripetal force, and so on. This, of course, characterizes exactly the well-recognized growth spiral of cities, sometimes labeled “energized crowding” in urban studies [96,97], but it can occur in just the same way in villages of modest size [16,98,99]. This may in fact be an accurate description of the dynamics of exaggerated growth that characterized Jenné-Jeno and Çatalhöyük, especially given the lack of centralized political organization or ritual integration that archaeologists generally expect to see in communities of their size.

The more usual pattern, though, most visible in Oaxaca, Barinas, the Alto Magdalena, and the Western Liao Valley, is that variation in the degree of nucleation of early settled communities led to variation in the degree of integration of local economies, which in turn led to variation in forms of early chiefdom organization. This leaves us with the question of how the social trajectories we have looked at got on different pathways—what led to variation in the degree of settlement nucleation in the first place?

One potential source of variation in settlement nucleation lies in the fundamental dynamics of early settled agriculture. We have, for example, argued elsewhere [87] that nucleated villages in Oaxaca formed in response to strategies for mitigating agricultural risk that required cooperation (and thus interaction) among groups of substantial size—interaction that drew people together into compact villages. In contrast, no interaction between households was called for to mitigate agricultural risk in the Alto Magdalena or the Western Liao Valley, because agricultural risk was minimal in the Alto Magdalena and because the best strategies in the Western Liao involved independent action by individual households. Connections between nucleation and agricultural risk mitigation strategies are beginning to recur across a broader comparison of chiefdom trajectories [93], though we doubt that risk mitigation is the only, or even the principal, factor to have influenced early settlement nucleation in all regions. It is not at all clear that agricultural risk mitigation is connected to settlement nucleation in Barinas or at Jenné-Jeno and Çatalhöyük, although it might have been.

Whether settlement nucleation is produced by agricultural risk mitigation strategies or other factors, it is clear that the degree of nucleation either facilitated or impeded the development of productive differentiation and the integration of early local economies. In turn, this development—often in combination with other factors—shaped the varied organizational forms of early chiefdoms. Our results here converge with and complement those of other studies that emphasize the role of local interaction in shaping human societies [1,74,83,91–93,100–102]. If the forces that produce social change are generated in the matrix of local interaction between households, then it is worth continuing to explore the role that household spacing played in shaping that change.

## Methodological implications

The analyses that the arguments developed in this paper rely on call for particular kinds of data not always available from archaeological field research. Availability of appropriate data was, in fact, among the reasons for choosing the cases that we worked with (and that made it impossible to work with others). Analyses of household artifact assemblages like those above clearly depend on fairly detailed typological or descriptive information about the nature of artifacts recovered. This sort of information is a staple of archaeological reports and has been for decades, if not centuries. But this information, in and of itself, is not enough.

Information on household artifact assemblages for analyses like those above can come from extensive excavations of house structures and associated trash deposits. But knowledge of house structures is, perhaps paradoxically, not really necessary; it is the accumulated trash from archaeological deposits of all kinds that is the best source of artifact samples. In contrast to artifacts found directly on house floors, such samples are both time-averaged, providing a picture of long-term activity patterns, and contain large numbers of artifacts, yielding statistical confidence in analytical results. Of the six artifact datasets analyzed above, only one (Çatalhöyük) came from excavation of residential architecture. For Oaxaca, Barinas, and Jenné-Jeno, the artifacts came primarily from small stratigraphic tests scattered so widely across a residential area that each test could be taken to represent trash associated with a different household location. These excavations only incidentally (for our purposes) revealed remains of house structures. Artifacts from Mesitas were recovered from numerous small, closely-spaced shovel probes, grouped into clusters that each represented a high-density trash accumulation from one or two households. The same approach recovered the Western Liao artifact samples, except that no excavation at all was needed; in these deflated sites, intensive surface collection of artifacts revealed high-density clusters similar to those at Mesitas and produced large samples of artifacts.

The cases studied here also met an important criterion of spatial resolution. In order to monitor variation between households, the recovery of data must make it possible to discriminate between artifact assemblages at a spatial scale corresponding roughly to individual households. This means that an artifact collection across an area larger than that covered by one or two households (typically no more than 10–20 m across) will not do because it averages together artifacts that represent the activities of too many households. By the same token, artifact collections within an area likely representing only one or two households must not be separated into different assemblages for analysis.

Recovering household artifact assemblages from shovel probes or surface collections will undoubtedly seem like a dubious proposition to many archaeologists. In both the Alto Magdalena and the Western Liao Valley, however, sites are shallow and artifacts of all periods are well represented on the surface. Houses were spaced far enough apart to create easily recognizable areas of high-density artifacts for delineating different household units. Large samples of artifacts were easily and quickly obtained from each household unit—samples that are likely to better represent each household unit because shovel probes and surface collections are broadly spread across its entire area. And the efficiency of shovel probes or surface collections for this task makes it possible to sample a much larger number of households in the settlement. As a consequence of this last observation, there were more households in the samples from the Alto Magdalena and Western Liao communities than in any other sample of households we studied.

It is not a concern here that the artifact assemblages analyzed were recovered from stratigraphic excavations, shovel tests, and surface collections because materials recovered in these different ways were not compared to each other. It was instead the index of productive differentiation that was compared between communities, and this was determined for each community on the basis of artifacts recovered by the same technique.

Even in a study focused on household activities, then, the issue of sampling arises at multiple scales. At the scale of the individual household, the question is how activities can best be represented by a sample of artifacts from each household unit. At the larger scale of the community, the question is how the differentiation of household activities can best be represented by a sample of households from the community. At the still larger scale of the region, the question is how typical for the region the patterns delineated at one or a few communities are. At this scale, too, the issue is one of sampling: how to characterize the thing that needs to be characterized (household, community, or region) by analyzing only a part of it. Obviously, we all feel better if our conclusions are based on a good-sized sample, but just what “good-sized” means in the context of samples of households or artifacts is usually discussed in only the vaguest of terms. It is possible to put a finer point on it, and failing to do so can impose severe limitations on the utility of laboriously-collected datasets. As with all methodological issues, the determination of how big is a good-sized sample depends on what research questions have been posed and what kinds of data analysis will be needed to answer them.

The analyses of household assemblages above depend on reliable proportions and ratios of different types of artifacts in each household assemblages—“reliable” in the sense that the impact of the random noise prevalent in small samples is minimal. For instance, a sample of 200 artifacts from a household allows for calculation of proportions of different things in that assemblage with an error range no greater than ±6% for 90% confidence. This signifies a level of random noise compatible with multivariate analysis and provides a way to think concretely, even if approximately, about what a good-sized artifact sample is. An even larger sample would be better, but not very much better—at least not for our purposes here. At around 200 artifacts per household one is getting to the point of diminishing returns in terms of the benefit of increasing the sample size. Likewise, the risk of pursuing patterns in randomness increases with smaller artifact samples, but gradually. The inclusion of some households for which the artifact samples are less than 200 will not damage a multivariate analysis much. But a multivariate analysis based on assemblage samples mostly consisting of 20 or 30 artifacts per household will not be reliable.

At the next larger scale, a good-sized sample of households from a community has a somewhat different meaning. It does not involve proportions or ratios of households; instead, the sample of households must represent the range of diversity of artifact assemblages reasonably well. In the early stages of productive differentiation, the unusual assemblages the reveal it would not likely be as rare as, say, 5% of the households in a community. But even if the proportion was that low, a sample of 50 households would make us more than 90% confident of finding at least one of those assemblages. Fifty, then, would seem to be a useful practical definition of a good-sized sample of households. Two of the datasets analyzed above (Mesitas and Upper Daling) were samples of 50 or more households. The Fábrica San José sample was only 10, but it comprised essentially the entire community. It thus provides a comprehensive view of the range of assemblage variation in this small village, and substantial productive differentiation was, indeed, detected. The Çatalhöyük sample was only 15, all from one small sector of the site, and this does raise some worrisome issues. A failure to detect substantial productive differentiation, however, was not among them; this sample, as small as it was, did reflect substantial productive differentiation, and a larger sample is likely to show even more.

At the still larger regional scale, it is not so much a question of acquiring a good-sized sample of communities, since, as is often the case, we took a single community as typical of each region. Questions of what would be labeled *bias* in statistics were addressed above by considering ways in which some communities would be more typical of their regions than others (here, B97 rather than Gaván in Barinas, or Fábrica San José rather than San José Mogote in Oaxaca, as discussed above).

## Materials and methods

No permits were required for the described study, which complied with all relevant regulations. All statistical analyses were performed using SYSTAT 13.1. The hypothetical examples of household spacing in Figs 2–4 were created so as to clearly illustrate abstract principles of distance and interaction. They were based on contemporary settlements whose residential densities approximate those of the six archaeological communities whose household artifact assemblages were analyzed. All were patterned after communities found in satellite imagery in different parts of the world so as to incorporate some of the idiosyncrasies of real-world household spacing. Households were subtracted from or added to these communities so that each example had exactly 250 households for clarity of comparison. Household artifact assemblage data came from published sources; details of how these data were manipulated are in Supporting Information (S1 Text).

## Supporting information

S1 Table Household assemblage artifact data. The artifact data that were the input for the multidimensional scaling analyses are provided in a single spreadsheet file with six worksheets, one for each site. These worksheets contain the ratios and proportions that were the variables for each household unit. The values of the ratios and proportions were derived transparently from the tables of counts previously published by the various authors, with one exception. For Jenné-Jeno, the combining of stratigraphic units into household units was a complicated consolidation of the published tables of counts, so the consolidated counts also appear for Jenné-Jeno on a seventh worksheet in the file.
(XLS)

S2 Table Multidimensional scaling configurations. The results of the multidimensional scaling analyses that were, in turn, the input for the *k*-means analyses are provided in a single spreadsheet file with six worksheets, one for each site, with the coordinates in two dimensions for the points in the scaling configuration. A final column contains the cluster number for each case in the *k*-means analysis (the central cluster is always No. 1).
(XLS)

S1 Text Sources of household assemblage artifact data. Information on each of the six sites whose household assemblage artifact data were the input for the multidimensional scaling analyses, along with a list of the variables used in those analyses, is provided in a single document.
(PDF)
