# Supplementary material for: Local economies and household spacing in early chiefdom communities
Source: PLoS One. 2021 May 27;16(5):e0252532. doi: 10.1371/journal.pone.0252532 (PMC8158874; doi:10.1371/journal.pone.0252532)
Supplement: S1 Text — Information on each of the six sites whose household assemblage artifact data were the input for the multidimensional scaling analyses, along with a list of the variables used in those analyses, is provided in a single document. (PDF) [file pone.0252532.s003.pdf]

# Local Economies and Household Spacing in Early Chiefdom Communities

## Sources of Household Artifact Assemblage Data

*Fábrica San José Data*

Household artifact assemblage data analyzed for the Valley of Oaxaca came from the Late Guadalupe phase occupation at the site of Fábrica San José (Drennan 1976). Stratigraphic tests (1 by 2 m) were spread across the entire site; some of them were later expanded to larger exposures. Remains of house floors, hearths, middens, and pit features were often encountered in the deeply stratified deposits (more than 3 m in some places). The 10 household units that became the cases in the multidimensional scaling grouped nearby excavation units together as originally done by Drennan. Artifact counts were the totals of all artifacts encountered in Late Guadalupe phase deposits in all excavations included in each household unit. The variables used for characterizing the contents of the household assemblages were the following ratios and proportions (underscored words are column headings in the spreadsheet **S1_Table.xls**):

- total number of flaked stone artifacts / total number of sherds
- total number of obsidian flaked stone artifacts / total number of flaked stone artifacts
- total number of shell fragments / total number of sherds
- total number of mica fragments / total number of sherds
- total number of polishing pebbles / total number of sherds
- total number of pieces of red ochre / total number of sherds
- total number of sherd disks / total number of sherds
- total number of bone needles / total number of sherds
- total number of bone awls / total number of sherds
- total number of bone gouges / total number of sherds
- total number of salt encrusted sherds / total number of sherds

*B97 Data*

Household artifact assemblage data analyzed for the Barinas region came from the Late Gaván phase occupation at the site of B97 (Spencer and Redmond 2014). Stratigraphic tests (1 by 2 m) were spread across the entire site. Hearths, middens, pit features, and burned daub were encountered in deposits usually less than 1 m deep. The tests were generally located 50 m or more apart, so each was taken to represent a separate household unit except for 56 and 61, which were combined because they were adjacent to each other. Tests that yielded very few artifacts were omitted from analysis. Each of the 23 household artifact assemblages that make up the cases in the multidimensional scaling consists of all artifacts recovered from the corresponding test. Following Spencer and Redmond, all artifacts are taken to represent the Late Gaván phase. The variables used for characterizing the contents of the household assemblages were the following ratios and proportions (underscored words are column headings in the spreadsheet **S1_Table.xls**):

- total number of reworked sherds / total number of sherds
- total number of grooved sherds / total number of sherds
- total number of sherd disks / total number of sherds
- total number of notched sherds / total number of sherds
- total number of kiln wasters / total number of sherds
- total number of lithic artifacts / total number of sherds
- total number of chert artifacts / total number of lithic artifacts
- total number of utilized flakes / total number of lithic artifacts
- total number of cores and core tools / total number of lithic artifacts
- total number of flakes / total number of lithic artifacts
- total number of blades / total number of lithic artifacts
- total number of reused flakes / total number of lithic artifacts
- total number of primary utilized flakes / total number of lithic artifacts
- total number of manos and metates / total number of lithic artifacts

- total number of mortars and pestles / total number of lithic artifacts
- total number of axes and celts / total number of lithic artifacts
- total number of polishing pebbles / total number of lithic artifacts

*Mesitas Data*

Household artifact assemblage data analyzed for the Alto Magdalena came from the Regional Classic period occupation at Mesitas (González Fernández 2007a, 2007b). A total of 3,000 shovel probes (40 by 40 cm) at a spacing of 5 m were excavated along transects through occupied areas as determined by regional survey. Clusters of shovel probes with high artifact densities were taken to represent household units, and 2,057 additional shovel probes were excavated within these clusters to obtain a larger sample of artifacts from each household unit. The 75 cases in the multidimensional scaling represent household units of the Regional Classic period as defined by González. The ceramic sherds for each household unit are the total of all Regional Classic period sherds recovered from the shovel probes assigned to that household unit. Non-ceramic artifacts from a shovel probe cannot be individually assigned to a period, so they represent the total number of artifacts of each class from the shovel probes assigned to that household unit. The variables used for characterizing the contents of the household assemblages were the following ratios and proportions (underscored words are column headings in the spreadsheet **S1_Table.xls**):

- total number of flaked stone artifacts / total number of Regional Classic sherds
- total number of obsidian flaked stone artifacts / total number of flaked stone artifacts
- total number of chert flaked stone artifacts / total number of flaked stone artifacts
- total number of slate flaked stone artifacts / total number of flaked stone artifacts
- total number of basalt flaked stone artifacts / total number of flaked stone artifacts
- total number of cores / total number of flaked stone artifacts
- total number of flakes / total number of flaked stone artifacts
- total number of scrapers / total number of flaked stone artifacts
- total number of pieces of debitage / total number of flaked stone artifacts
- total number of manos and metates / total number of Regional Classic sherds
- total number of stone spheres / total number of Regional Classic sherds
- total number of polishing pebbles / total number of Regional Classic sherds
- total number of adzes / total number of Regional Classic sherds
- total number of axes / total number of Regional Classic sherds
- total number of chisels / total number of Regional Classic sherds
- total number of other ground stone artifacts / total number of Regional Classic sherds
- total number of quartz fragments / total number of Regional Classic sherds
- total number of notched stone disks / total number of Regional Classic sherds
- total number of stone cylinders / total number of Regional Classic sherds
- total number of kiln wasters / total number of Regional Classic sherds

*Upper Daling Data*

Household artifact assemblage data analyzed for the Western Liao Valley came from three zones of Hongshan period occupation (Dongshanzui, Sanjia, and Erbuchi) in the Upper Daling Valley (Drennan et al. 2017; Peterson et al. 2017). In these deflated sites, the vast majority of artifacts are at or near the surface, and hot spots of high-density surface artifacts from garbage deposits represent the remains of households, as confirmed by stratigraphic excavation, magnetometry, and abundant burned daub. In each hot spot, all artifacts were collected from the surface by grid square (5 by 5 m), and the upper 5 cm of soil was screened for additional artifact recovery. The 50 cases in the multidimensional scaling represent household units as defined by Drennan, Peterson, et al., and the scaling itself is the first two dimensions of the three-dimensional lithic scaling previously published. The data include all artifacts recovered in the intensive surface collection. Although there is no stratigraphic information about the samples, the non-ceramic artifacts can be counted on as a good representation of the Hongshan period, since ceramics of other periods were present in only trivial quantities in these locations. The variables used for characterizing the contents of the household assemblages were the following ratios and proportions (underscored words are column headings in the spreadsheet **S1_Table.xls**):

- total number of lithic tools of high-quality raw material / total number of lithic tools
- total number of ground stone tools / total number of lithic tools
- total number of grooved abraders / total number of lithic tools
- total number of awls and drills / total number of lithic tools
- total number of axes and adzes / total number of lithic tools
- total number of blades / total number of lithic tools
- total number of retouched flakes / total number of lithic tools
- total number of unifacial scrapers / total number of lithic tools
- total number of chopping tools / total number of lithic tools
- total number of blade cores / total number of lithic tools
- total number of flake cores / total number of lithic tools
- total number of projectile points / total number of lithic tools
- total number of blanks and preforms / total number of lithic tools
- total number of scrapers, retouched blades, and flakes with acute edge angles (25–44°) / total number of lithic tools
- total number of scrapers, retouched blades, and flakes with obtuse edge angles (45–65°) / total number of lithic tools
- total number of complete unretouched flakes / total number of pieces of debitage
- total number of broken unretouched flakes / total number of pieces of debitage
- total number of pieces of lithic shatter / total number of pieces of debitage
- total weight of debitage (g) / total number of pieces of debitage

*Çatalhöyük Data*

Household artifact assemblage data analyzed for the Anatolian Plain came from excavations at Çatalhöyük East, representing Late Pre-Pottery Neolithic B, Pre-Pottery Neolithic C, and the beginnings of Late Neolithic (Wright 2014). The artifacts were recovered from house floors in the process of exposing the architectural remains of the dwellings of the 15 household units that became the cases in the multidimensional scaling. Household units that yielded very few artifacts were omitted from analysis. Following Hodder and Pels (2010:163), all houses were taken to be domestic in nature. Allocation of artifacts to household units follows Wright. The variables used for characterizing the contents of the household assemblages were the following ratios and proportions (underscored words are column headings in the spreadsheet **S1_Table.xls**):

- total number of quern fragments / total number of ground stone artifacts
- total number of pieces of debitage / total number of ground stone artifacts
- total number of handstones / total number of ground stone artifacts
- total number of palettes / total number of ground stone artifacts
- total number of axes and celts / total number of ground stone artifacts
- total number of hammerstones / total number of ground stone artifacts
- total number of abraders / total number of ground stone artifacts
- total number of fragments of pigment / total number of ground stone artifacts
- total number of polishers / total number of ground stone artifacts
- total number of axe/celt preforms / total number of ground stone artifacts
- total number of pieces of miscellaneous worked stone / total number of ground stone artifacts
- total number of abrading slabs / total number of ground stone artifacts
- total number of abrader-knives / total number of ground stone artifacts
- total number of sanders / total number of ground stone artifacts
- total number of complete querns / total number of ground stone artifacts
- total number of quern roughouts / total number of ground stone artifacts
- total number of sanding slabs / total number of ground stone artifacts
- total number of figurine-related stone pieces / total number of ground stone artifacts
- total number of stone balls / total number of ground stone artifacts
- total number of pestles / total number of ground stone artifacts
- total number of grooved abraders / total number of ground stone artifacts
- total number of shaft straighteners / total number of ground stone artifacts

- total number of choppers / total number of ground stone artifacts
- total number of cores / total number of ground stone artifacts
- total number of worktable-anvils / total number of ground stone artifacts
- total number of stone vessels (general) / total number of ground stone artifacts
- total number of polishing slabs / total number of ground stone artifacts
- total number of stone hoes / total number of ground stone artifacts
- total number of maceheads / total number of ground stone artifacts
- total weight of ground stone artifacts / total number of ground stone artifacts
- total number of other perforated stones / total number of ground stone artifacts
- total number of stone bowls / total number of ground stone artifacts
- total number of stone platters / total number of ground stone artifacts

*Jenné-Jeno Data*

Household artifact assemblage data analyzed for the Middle Niger Delta came from excavations at the site of Jenné-Jeno (McIntosh, ed., 1995). Stratigraphic tests of various sizes were spread across the site; some were later expanded. Houses, walls, hearths, middens, pits, iron-smelting furnaces, and other features were encountered in deeply stratified deposits (up to some 6 m deep). Each of the 31 household artifact assemblages that make up the cases in the multidimensional scaling consists of all artifacts recovered from all deposits of a single phase (Phase 3 or Phase 4) in a particular test. Thus the artifacts from the Phase 3 deposits in a test unit might comprise one case and the artifacts from the Phase 4 deposits in that test unit might comprise another case. Deposits that were dated only as Phase 3–4 could not, of course, be separated in this way into two cases. The variables used for characterizing the contents of the household assemblages were the following ratios and proportions (underscored words are column headings in the spreadsheet **S1_Table.xls**):

- total number of iron tools / total number of sherds
- total number of crucibles and crucible fragments / total number of sherds
- total weight of slag / total number of sherds
- total number of furnace fragments / total number of sherds
- total number of spindle whorls / total number of sherds
- total number of ceramic and stone weights / total number of sherd

*References*


Drennan, Robert D.

1976 *Fábrica San José and Middle Formative Society in the Valley of Oaxaca*. Memoirs of the Museum of Anthropology, University of Michigan, No. 8.

Drennan, Robert D., Christian E. Peterson, Lu Xueming, and Li Tao

2017 Hongshan Households and Communities in Neolithic Northeastern China. *Journal of Anthropological Archaeology* 47:50–71.

González Fernández, Víctor

2007a *Prehispanic Change in the Mesitas Community: Documenting the Development of a Chiefdom's Central Place in San Agustín, Huila, Colombia / Cambio prehispánico en la comunidad de Mesitas: Documentando el desarrollo de la comunidad central en un cacicazgo de San Agustín, Huila, Colombia.* University of Pittsburgh Memoirs in Latin American Archaeology, No. 18.

2007b *Mesitas Community Dataset*. Comparative Archaeology Database, University of Pittsburgh. URL: <www.cadb.pitt.edu>.

Hodder, Ian, and Peter Pels

2010 History Houses: A New Interpretation of Architectural Elaboration at Çatalhöyük. In *Religion in the Emergence of Civilization: Çatalhöyük as a Case Study*, Ian Hodder (ed.), pp. 163-186. Cambridge: Cambridge University Press.

McIntosh, Susan Keech (ed.)

1995 *Excavations at Jenné-Jeno, Hambarketolo, and Kaniana (Inland Niger Delta, Mali), the 1981 Season*. University of California Publications, Anthropology, Vol. 20. Berkeley: University of California Press.

Peterson, Christian E., Lu Xueming, Robert D. Drennan, and Zhu Da
2017 *Upper Daling Region Hongshan Household and Community Dataset*. Comparative Archaeology Database, University of Pittsburgh. URL: <www.cadb.pitt.edu>.
Spencer, Charles S., and Elsa M. Redmond
2014 *A Pre-Hispanic Chiefdom in Barinas, Venezuela: Excavations at Gaván-Complex Sites*. American Museum of Natural History Anthropological Papers, No. 100.
Wright, Katherine I.
2014 Domestication and Inequality? Households, Corporate Groups and Food Processing Tools at Neolithic Çatalhöyük. *Journal of Anthropological Archaeology* 33:1–33.
